# BLISTER-regulated vegetative growth is dependent on the protein kinase domain of ER stress modulator IRE1A in *Arabidopsis thaliana*

**DOI:** 10.1371/journal.pgen.1008563

**Published:** 2019-12-23

**Authors:** Zheng-Hui Hong, Tao Qing, Daniel Schubert, Julia Anna Kleinmanns, Jian-Xiang Liu

**Affiliations:** 1 State Key Laboratory of Genetic Engineering, School of Life Sciences, Fudan University, Shanghai, China; 2 State Key Laboratory of Plant Physiology and Biochemistry, College of Life Sciences, Zhejiang University, Hangzhou, China; 3 Plant Developmental Epigenetics, Heinrich Heine University Düsseldorf, Düsseldorf, Germany; 4 Epigenetics of Plants, Freie Universität Berlin, Berlin, Germany; Peking University, CHINA

## Abstract

The unfolded protein response (UPR) is required for protein homeostasis in the endoplasmic reticulum (ER) when plants are challenged by adverse environmental conditions. Inositol-requiring enzyme 1 (IRE1), the bifunctional protein kinase / ribonuclease, is an important UPR regulator in plants mediating cytoplasmic splicing of the mRNA encoding the transcription factor bZIP60. This activates the UPR signaling pathway and regulates canonical UPR genes. However, how the protein activity of IRE1 is controlled during plant growth and development is largely unknown. In the present study, we demonstrate that the nuclear and Golgi-localized protein BLISTER (BLI) negatively controls the activity of IRE1A/IRE1B under normal growth condition in *Arabidopsis*. Loss-of-function mutation of *BLI* results in chronic up-regulation of a set of both canonical UPR genes and non-canonical UPR downstream genes, leading to cell death and growth retardation. Genetic analysis indicates that *BLI*-regulated vegetative growth phenotype is dependent on IRE1A/IRE1B but not their canonical splicing target *bZIP60*. Genetic complementation with mutation analysis suggests that the D570/K572 residues in the ATP-binding pocket and N780 residue in the RNase domain of IRE1A are required for the activation of canonical UPR gene expression, in contrast, the D570/K572 residues and D590 residue in the protein kinase domain of IRE1A are important for the induction of non-canonical UPR downstream genes in the *BLI* mutant background, which correlates with the shoot growth phenotype. Hence, our results reveal the important role of IRE1A in plant growth and development, and BLI negatively controls IRE1A’s function under normal growth condition in plants.

## Author summary

When unfolded or misfolded proteins are accumulated in the ER, a much conserved response, called the unfolded protein response (UPR), is elicited to lighten the load of unfolded proteins in the ER by bringing the protein-folding and degradation capacities into alignment with the protein folding demands. However, over-activation of the UPR pathways under normal growth conditions affects plant growth and development. The bifunctional protein kinase / ribonuclease protein IRE1 is important for UPR gene regulation, but how IRE1’ protein activity is tightly controlled in plants is currently unknown. Here we report that BLISTER (BLI) negatively controls the IRE1’s function under normal growth condition in *Arabidopsis*. Through genetic analysis, our results also provide novel insights into how the protein kinase domain and ribonuclease domain contribute to the function of IRE1A in downstream gene expression.

## Introduction

Protein folding in the ER is a fundamental process in eukaryotic cells. Protein folding demands on the secretory pathway escalate constantly during different developmental stages and environmental conditions while protein folding capacity is limited depending on ER chaperones, oxidoreductases, N-glycosylation etc. [1,2]. When protein folding demands exceed the protein folding capacity in the ER, unfolded or misfolded proteins accumulate, triggering the UPR pathway in order to recover protein homeostasis in the ER [3]. Like yeast and mammalian cells, plant cells are also equipped with a set of membrane-associated transcription factors for sensing/transducing ER stress signals, although the sequence similarities among these factors are modest [4–14]. These membrane-associated transcription factors are activated in a similar way to those in yeast and mammals. In Arabidopsis, both bZIP28 and bZIP60 are ER-membrane-associated basic Leucine Zipper (bZIP) transcription factors. Upon ER stress induced by chemicals or abiotic stresses such as heat stress, bZIP28 relocates from ER to Golgi, where it is subjected to proteolysis by Golgi-resident Site-2 Protease (S2P). This cleavage releases its cytoplasmic N-terminal region that contains a DNA-binding domain, a nuclear localization signal and a transcriptional activation domain for downstream stress responsive gene expression [4,15–20]. The activation of bZIP60 is distinct and requires unconventional splicing of bZIP60 mRNA executed by the ER-membrane-associated protein IRE1 containing a protein kinase domain and a ribonuclease (RNase) domain in its C-terminus. Under ER stress conditions, the activated IRE1 recognizes the double stem-loop structure on bZIP60 mRNA and splices out a 23-base nucleotides, which results in a reading frame shift and elimination of the transmembrane domain of encoded bZIP60 [10,14,21]. When the soluble form of bZIP60 enters the nucleus, it activates downstream ER stress responsive genes [8,22]. Beside specifically targeting *bZIP60* mRNA, IRE1 becomes more promiscuous under severe ER stress conditions, and attacks other mRNAs through a process called Regulated IRE1-Dependent Decay (RIDD), which is thought to be important for ER stress tolerance in *Arabidopsis* [23]. The UPR has paradoxical outputs, cytoprotective effects for protein homeostasis and cytotoxic effects to induce programmed cell death, depending on the intensity and duration of the stimulus that the organism, tissues or cells are receiving [1]. Over-activation of the UPR pathways affects plant growth and development. For example, constitutive over-expression of the activated form of bZIP28 in Arabidopsis induces UPR genes and results in delayed growth and development [4]. Recently, it was reported that mutation of the rice transcription factor SQUAMOSA PROMOTER-BINDING PROTEIN-LIKE 6 (SPL6) causes up-regulation of IRE1 expression and persistent UPR, leading to cell death and apical panicle abortion in rice [24]. However, how IRE1 activity is controlled under normal growth conditions in plants is currently unknown.

In the current study, we found that BLISTER (BLI) negatively regulates IRE1A function in Arabidopsis. Mutation of *BLI* results in the activation of IRE1A and up-regulation of both canonical and non-canonical UPR genes under normal growth conditions, however, the IRE1-bZIP60 pathway is not responsible for the vegetative growth retardation phenotype in the *BLI* mutant plants. Further studies demonstrated that the residues D570/K572 and D590 in the protein kinase domain of IRE1A are important for non-canonical UPR gene expression and growth inhibition, while the D570/K572 residues and N780 residue of IRE1A are required for its ribonuclease activity to induce canonical UPR downstream genes. Together, these results demonstrated the important role of IRE1A in growth and development, and revealed BLI as a new negative regulator of IRE1 in Arabidopsis.

## Results

### Mutation of *BLI* results in up-regulation of UPR genes and growth retardation phenotype under normal growth conditions

**Fig 1:**
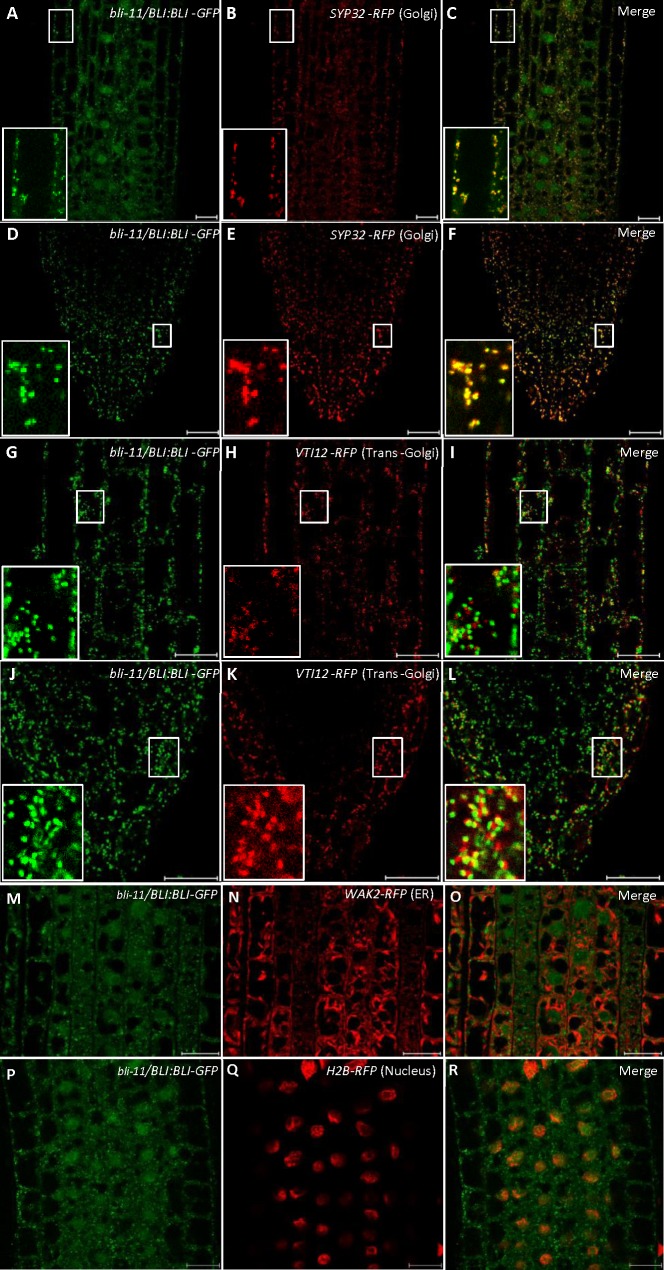
BLI protein localizes in Golgi and nucleus. **A-R**, Subcellular localization of BLI in *Arabidopsis bli-11* mutant plants. *BLI*:*BLI-GFP* was co-expressed with the Golgi marker *SYP32-RFP*, the trans-Golgi/early endosome marker *VTI12-RFP*, the ER marker *WAK2-RFP*, or the nucleus marker *H2B-RFP*. Root cells in the elongation zone (A-C, G-I, M-R) or meristematic zone (D-F, J-L) were observed under a confocal microscope and merged. Close-up pictures are shown in A-L. Scale bars are 20 μm.

Previously we identified a plant specific protein BLISTER (BLI), which interacts with the Polycomb-group (Pc-G) histone methyltransferase CURLY LEAF (CLF) and controls the expression of Pc-G target genes for cellular differentiation in *Arabidopsis thaliana* [25]. Mutations of *BLI* in two loss-of-function Arabidopsis mutant alleles (*bli-1* and *bli-11*) conferred growth retardation [26,27] and up-regulation of some canonical ER stress responsive genes under normal growth conditions, for examples, *BiP3*, *NSF*, *ERDJ3A*, *ERDJ3B* and *SARA1A* (S1A Fig). The spliced form of bZIP60 mRNA, encoding the activated nuclear form of bZIP60, was also dramatically up-regulated in both *bli-1* and *bli-11* mutant plants, while the unspliced bZIP60 mRNA was less up-regulated (S1A Fig), suggesting that bZIP60 is activated in the *BLI* mutant plants. However, the expression of neither *IRE1A* nor *IRE1B* is affected in the *BLI* mutant plants (S1A Fig). These results supports that UPR pathway is turned on when *BLI* is mutated in Arabidopsis, and BLI is a negative regulator of the ER stress response. Previously, we demonstrated that BLI-GFP was mainly observed in the nucleus with cytoplasmic signals in the transient expression system in tobacco (*N*. *benthamiana*) epidermal leaves [25]. Our previous analyses revealed that ER stress modulators, such as bZIP28, bZIP60, NAC062 and NAC089, localize to either ER membrane or plasma membrane in plants when they are inactive [4–7], so we re-checked the subcellular localization of BLI in stably transformed Arabidopsis plants. To do so, *BLI-GFP* was co-expressed with the ER marker *WAK2-RFP*, Golgi marker *SYP32-RFP* or the trans-Golgi/early endosome marker *VTI12-RFP*, or the nucleus marker *H2B-RFP* [28] in *Arabidopsis* plants. Confocal microscopy revealed that BLI-GFP not only localized to nucleus but also co-localized with SYP32-RFP but not with VTI12-RFP or WAK2-RFP in root cells (Fig 1A–1R). We conclude that BLI localizes to the Golgi and nucleus, and negatively regulates the expression of ER stress responsive genes in plants under normal growth condition.

### IRE1 Has UPR-independent roles in BLI-regulated plant growth and development

**Fig 2:**
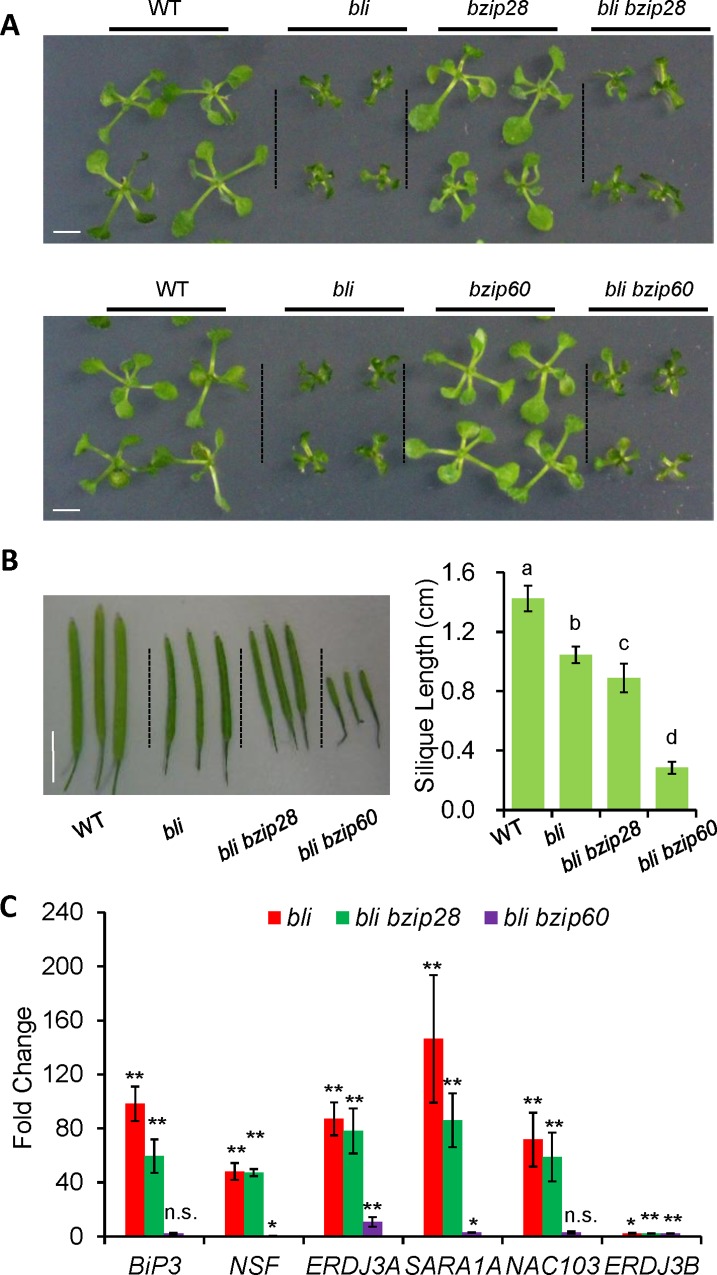
*bZIP60* mutation suppresses UPR gene expression but not shoot growth phenotype of *BLI* mutant plants. **A-B**, Phenotypic analysis. T-DNA mutant *bli* (*bli-1*) was crossed to either *bZIP28* mutant (*bzip28*) or *bZIP60* mutant (*bzip60*) to generate the respective double mutant plants. 2-week-old plant seedlings were photographed (A) and siliques lengths were measured later at reproductive stage (B). Error bars represent SD (n = 10). Letters above the bars in (B) indicate significant differences as determined by LSD test following ANOVA analysis (p<0.05). Bar = 5 mm. **C**, UPR gene expression analysis. Total RNA was exacted from 2-week-old plants for qRT-PCR. Fold change is the gene expression level in the mutants normalized to that in the WT, both of which were normalized to the expression of *ACTIN*. Asterisks indicate significance levels when comparing to the WT control in *t*-test. (*, p<0.05; **, p<0.01; n.s., not significant at p<0.05).

To investigate whether the growth phenotype of *BLI* mutant plants depends on ER stress response modulators, we crossed *bli* (*bli-1* hereafter) to either *bzip28* or *bzip60* and generated *bli bzip28* and *bli bzip60* double mutants. The *bzip28* and *bzip60* single mutants grew as normally as the WT (S2A and S2B Fig), and the expression of ER stress responsive genes such as *BiP3*, *ERDJ3A* and *SARA1A* in *bzip28* and *bzip60* single mutant seedlings was slightly changed (within one fold change) comparing to that in WT plants under normal growth conditions (S2C Fig). However, when crossed to the *bli* mutant, the *bli bzip28* double mutant resembled the *bli* single mutant phenotype in terms of shoot growth (Fig 2A) and silique length (Fig 2B). The *bli bzip60* double mutant also resembled the *bli* single mutant phenotype at the vegetative stage (Fig 2A), but the silique length in the *bli bzip60* double mutant was further reduced comparing to that in the *bli* single mutant (Fig 2B). These results suggest that the growth retardation phenotype of *bli* at the seedling stage does not depend on bZIP28 or bZIP60 alone. Further gene expression analysis showed that the expression of ER stress marker genes was not up-regulated in the *bli bzip60* double mutant seedlings, but still up-regulated in *bli bzip28* double mutant seedlings comparable to the inducing level in *bli* single mutant (Fig 2C). In our previous study, mutation of *bZIP28* reduces the up-regulation of *BiP3*, *ERDJ3A*, *ERDJ3B* and *SARA1* under ER stress condition [4]. *ERDJ3B* was considered as a bZIP28-specific downstream gene since knock-out *bZIP28* almost abolished the up-regulation of ERDJ3B during UPR in Arabidopsis [29]. However, the expression of *ERDJ3B* was only slightly up-regulated in *bli* mutant plants (Fig 2C and S1 Fig). The differences between *bli* and *bli bzip28* mutants in terms of up-regulation of *BiP3*, *NSF*, *ERDJ3A*, *SARA1* and *NAC103* were also very modest. Nevertheless, our results strongly suggest that bZIP60 is activated in *bli* mutant. As the up-regulation of the analyzed ER stress responsive genes in the *bli* mutant depends on bZIP60, and *bli bzip60* mutants are reminiscent of *bli* mutants at the seedling stage, it is likely that mis-expression of the analyzed ER stress genes does not contribute to the *bli* phenotype at seedling stage.

**Fig 3:**
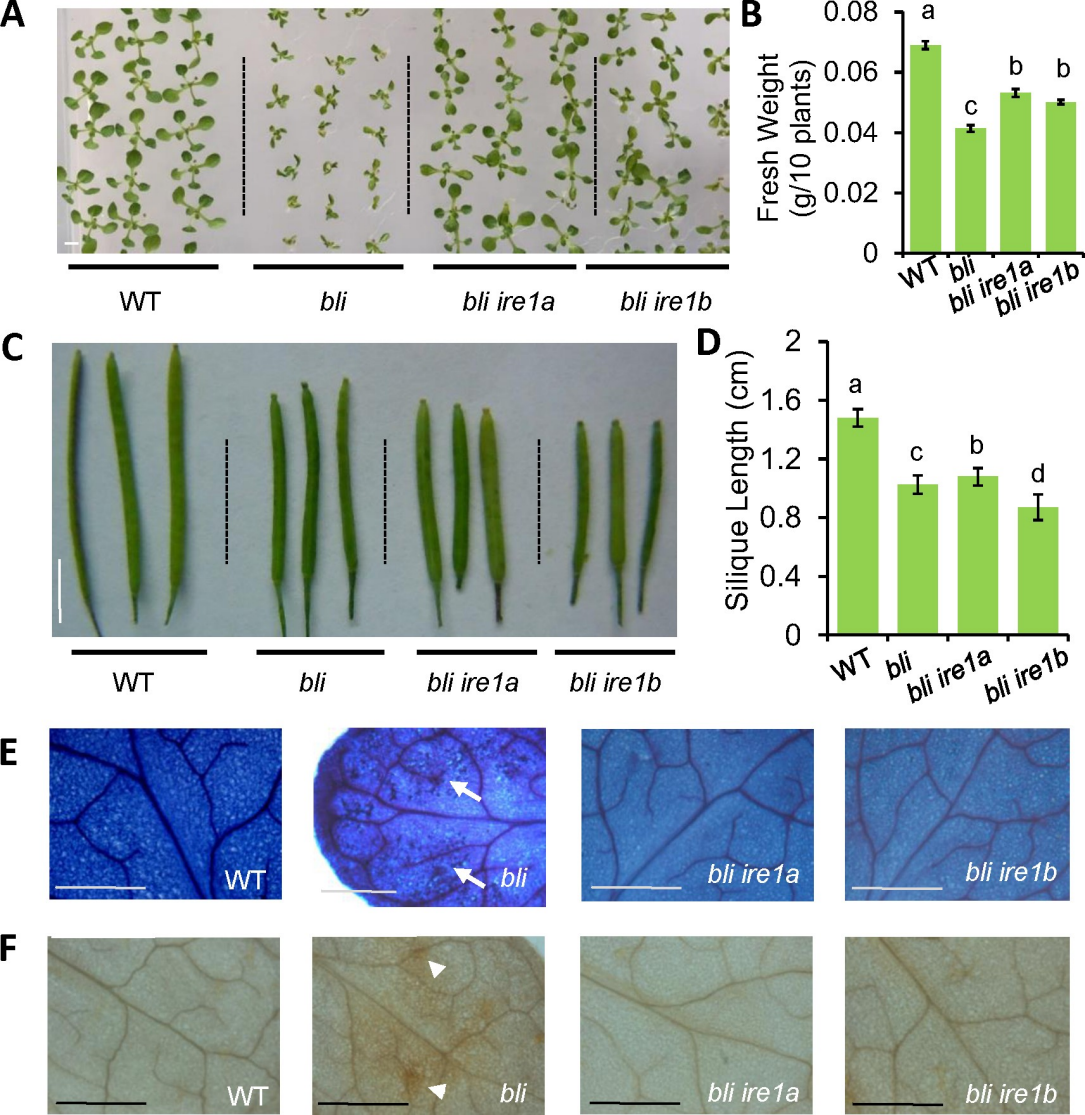
*IRE1* mutations suppress the shoot growth phenotype of *BLI* mutant plants. **A-D**, Phenotypic analysis. T-DNA mutant *bli* (*bli-1*) was crossed to either *IRE1A* mutant (*ire1a*) or *IRE1B* mutant (*ire1b*) to generate the respective double mutant plants. 2-weeks-old plants were photographed (A), weighted (B). Representative siliques were photographed (C) and siliques lengths were measurement at reproductive stage (D). Error bars represent SE (n = 3) in (B) and represent SD (n = 12) in (D). Letters above the bars indicate significant differences as determined by LSD test following ANOVA analysis (p< 0.05). Bar = 5 mm. **E-F**, Cell death and ROS accumulation analysis. 2-weeks-old plants were stained with trypan blue (E) or DAB (F). Arrows and arrow heads point to sites of cell death and ROS accumulation, respectively. Bar = 1 mm.

bZIP60 is an Arabidopsis ER-membrane-associated transcription factor which is activated by ER-localized IRE1A/IRE1B through unconventional splicing during the ER stress response [5,10,14]. IRE1A/IRE1B contain both protein kinase domains and RNase domains at the cytoplasm-facing side. The RNase activity of IRE1B is required for ER stress tolerance, whereas both protein kinase and RNase activity are required for normal root growth [30]. To analyze the genetic interaction of BLI and IRE1A/IRE1B, we crossed *bli* to either *ire1a* or *ire1b* mutant to generate the *bli ire1a* and *bli ire1b* double mutants (S3 Fig). The *ire1a* and *ire1b* single mutants did not have obvious phenotypes when compared to the WT (S2A and S2B Fig), and mutation of neither *IRE1A* nor *IRE1B* altered the expression of ER stress responsive genes under normal growth conditions (S2C Fig). However, mutation of either *IRE1A* or *IRE1B* largely suppressed the *bli* mutant phenotype at seedling stage in terms of seedling size (Fig 3A), but not at reproductive stage in terms of silique length (Fig 3C and 3D). However, the leaves of *bli ire1a* and *bli ire1b* plants were thinner than that of the WT plants. Meanwhile, the root growth retardation phenotype in *bli* was not suppressed in *bli ire1a* and *bli ire1b* mutants under normal growth condition (S4 Fig). Therefore, the fresh weight of whole plants was only partially suppressed in the *bli ire1a* and *bli ire1b* mutant plants (Fig 3B). Loss of *BLI* function resulted in cell death, as revealed by Trypan Blue staining (Fig 3E), and ROS accumulation, as revealed by DAB staining (Fig 3F). Mutation of either *IRE1A* or *IRE1B* also suppressed cell death and ROS level in the *bli* mutant seedlings (Fig 3E and 3F). To investigate whether BLI is involved in the ER stress response, the sensitivity of WT plants, *bli*, *ire1a*, and *ire1b* single mutants, as well as *bli ire1a* and *bli ire1b* double mutants were checked. In the presence of tunicamycin (TM), an N-glycosylation inhibitor specifically inducing misfolded protein accumulation in ER, in the growth medium, the *bli-1* mutant plants were very sensitive to ER stress in terms of shoot growth, leaf chlorosis and root growth, when compared to the WT, which could be partially rescued in the *bli ire1a* and *bli ire1b* double mutant plants (S4 Fig), although the expression of *BLI* is not affected by TM treatment (S2C Fig). Unfortunately, we could not obtain *bli ire1a ire1b* triple mutant plants. Arrested embryos from the *ire1a ire1b bli+/-* self-crossing siliques were observed (S5 Fig), suggesting that *bli ire1a ire1b* triple mutant is lethal. Taken together, these results demonstrate that BLI has both IRE1 dependent and independent roles in growth and development in Arabidopsis.

**Fig 4:**
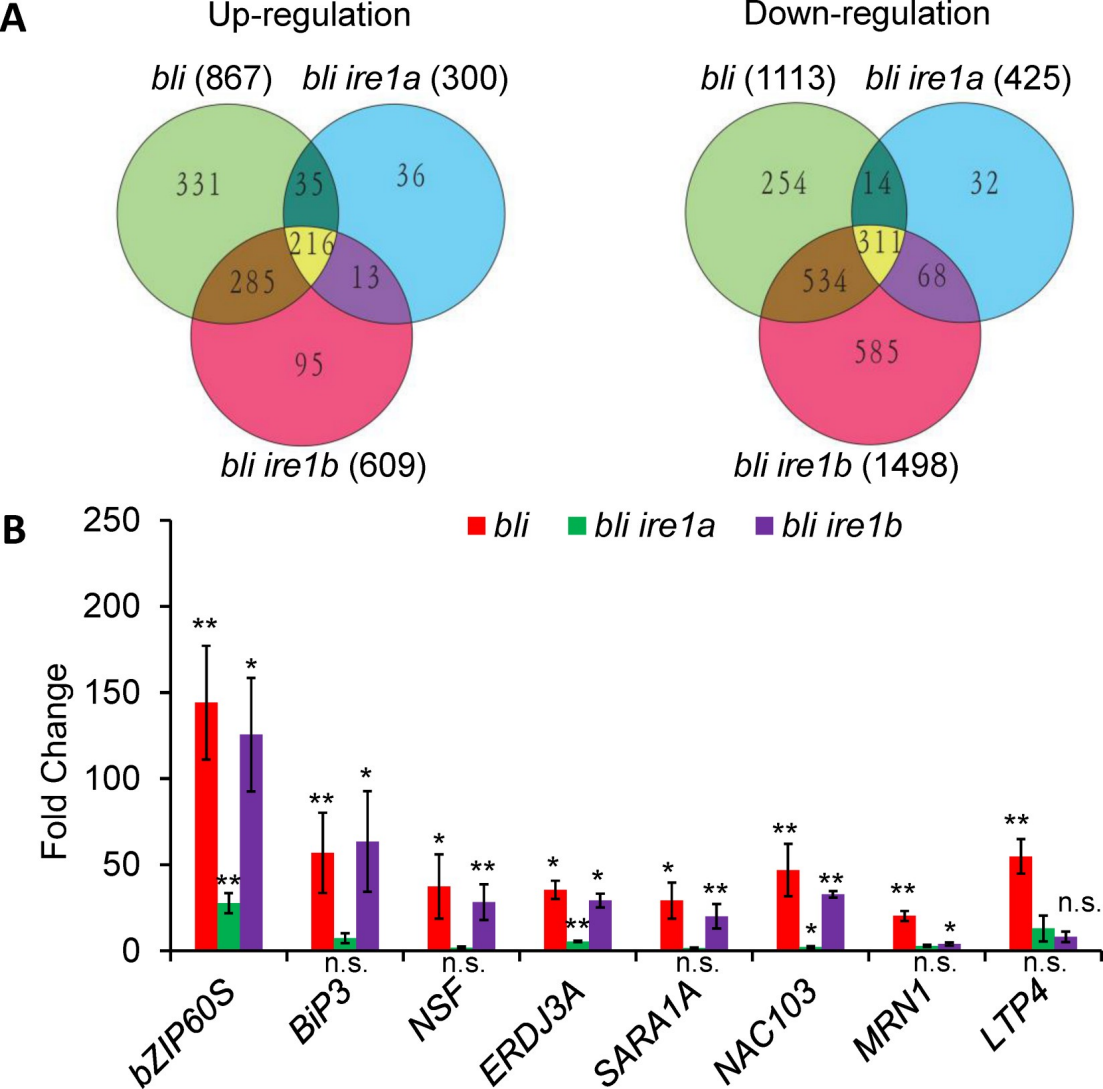
*IRE1A* mutation suppresses both canonical and non-canonical UPR gene expression in *BLI* mutant plants. **A-B**, Gene expression analysis. T-DNA mutant *bli* (*bli-1*) was crossed to either *IRE1A* mutant (*ire1a*) or *IRE1B* mutant (*ire1b*) to generate the respective double mutant plants. Total RNA was exacted for RNA-Seq analysis (A) and qRT-PCR (B). Up-regulated or down-regulated genes in the mutants relative to the wild-type plants were used to draw the Venn diagrams. Fold change is the gene expression level in the mutants normalized to that in the WT, both of which were normalized to the expression of *ACTIN*. Error bars represent SE (n = 3). Asterisks indicate significance levels when comparing to the WT control in *t*-test. (*, p<0.05; **, p<0.01; n.s., not significant at p<0.05). *bZIP60S*, spliced *bZIP60* transcript. Note that *bZIP60-S*, *BiP3*, *NSF*, *DERD3A*, *SARA1A* and *NAC103* are up-regulated while *MRN1* and *LTP4* are not up-regulated by canonical ER stress inducer tunicamycin.

To understand how IRE1 is involved in the *bli*-regulated shoot growth, we performed RNA-Seq analysis with WT, *bli*, *bli ire1a* and *bli ire1b* seedlings grown under normal growth conditions. Compared to the WT, 867 and 1113 genes were significantly up-regulated and down-regulated in the *bli* mutant, respectively (Fig 4A and S1C Fig). Among them, the expression of 359 up-regulated genes (i.e. 41.4%) and 599 down-regulated genes (i.e. 53.8%) was also affected by ER stress inducer tunicamycin (5 μg/ml, 12 hours) in WT plants (S1C Fig), further demonstrated that the canonical UPR pathway is activated in *bli* mutant seedlings. Interestingly, 331 up-regulated genes and 251 down-regulated genes in the *bli* mutant were not mis-regulated in *bli ire1a* or *bli ire1b* seedlings (Fig 4A and S1 Dataset). Surprisingly, up-regulation of canonical UPR responsive genes in the *bli* mutant was suppressed in the *bli ire1a* double mutant but not in *bli ire1b* double mutants, whereas the up-regulation of non-canonical ER stress responsive genes such as *MRN1* and *LTP4* was suppressed in both the *bli ire1a* and *bli ire1b* mutant (Fig 4B and S1 Dataset). This suggests that the RNase activity of IRE1A is more activated than that of IRE1B in *bli* mutant plants, or the RNase activity of IRE1B is dependent on IRE1A in *bli* mutant plants under normal growth condition. Nevertheless, our results show that both IRE1A and IRE1B, but not bZIP60, are required for the shoot growth phenotype in *bli* mutant, and the *bli* shoot growth phenotype is probably attributed to the mis-expression of non-canonical UPR genes.

### Residues in the protein kinase domain of IRE1A are important for BLI-regulated vegetative growth

**Fig 5:**
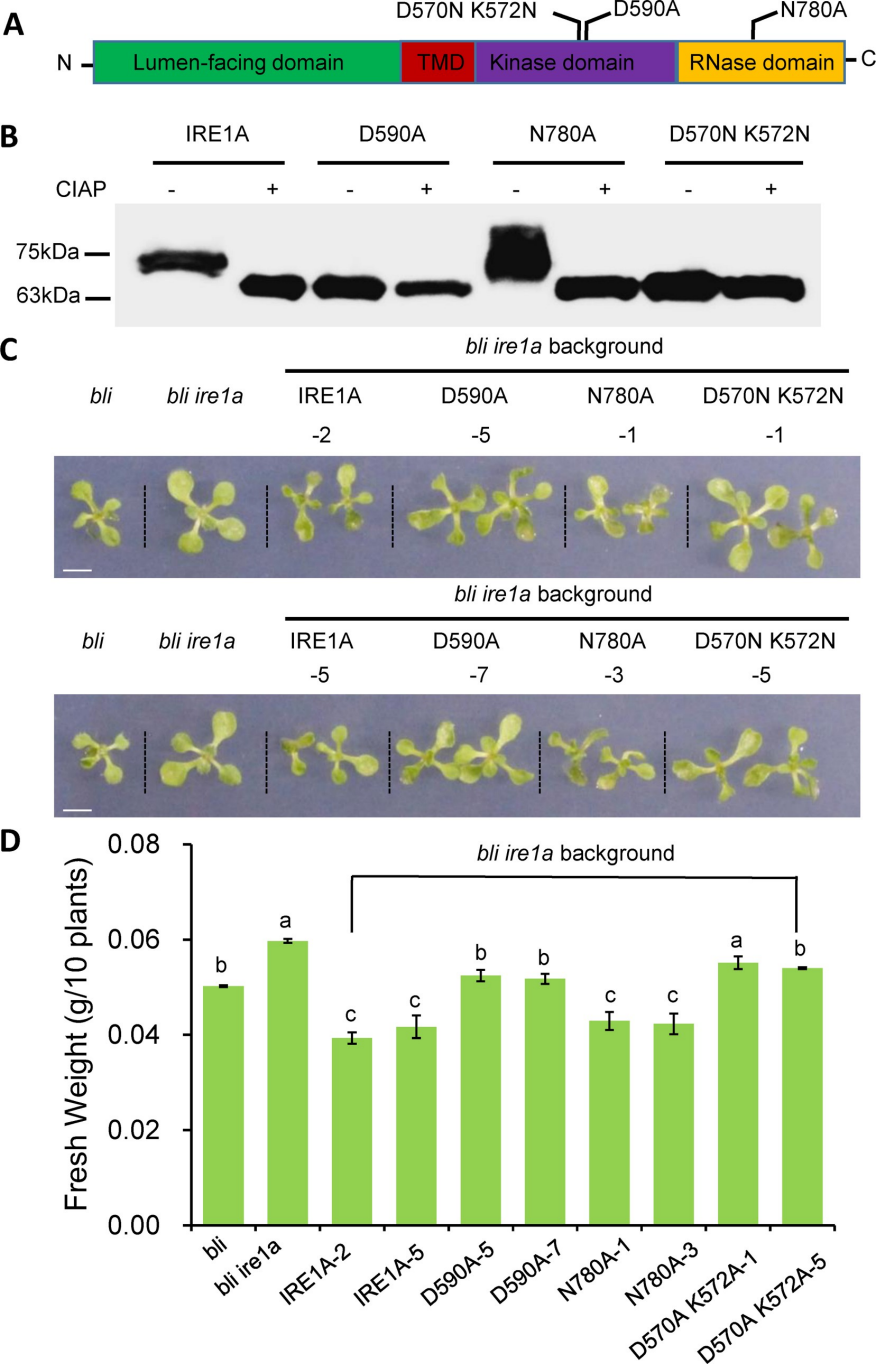
Residues in the protein kinase domain of IRE1A is required for the shoot growth phenotype in *BLI* mutant plant background. **A-B**, Site-specific mutagenesis analysis of the IRE1A auto-phosphorylation. Domain structure of IRE1A is shown in (A) with the mutated amino acids highlighted. The wild-type form and various mutated forms of IRE1A were expressed in *E*. *coli* and purified proteins were treated with CIAP for de-phosphorylation assays. Western blotting analysis was done to detect the molecular weight shifts. **C-D**, Genetic complementation analysis. T-DNA mutant *bli* (*bli-1*) was crossed to either *IRE1A* mutant (*ire1a*) or *IRE1B* mutant (*ire1b*) to generate the respective double mutant plants. The wild-type form and various mutated forms of IRE1A were expressed the *bli ire1a* double mutant background and 2-weeks-old T3 transgenic plants were photographed (C) and fresh weight were measured (D). Error bars represent SE (n = 3). There were at least 10 plants for each of the three biological replicates. Letters above the bars indicate significant differences as determined by LSD test following ANOVA analysis (p<0.05). Bar = 5 mm.

**Fig 6:**
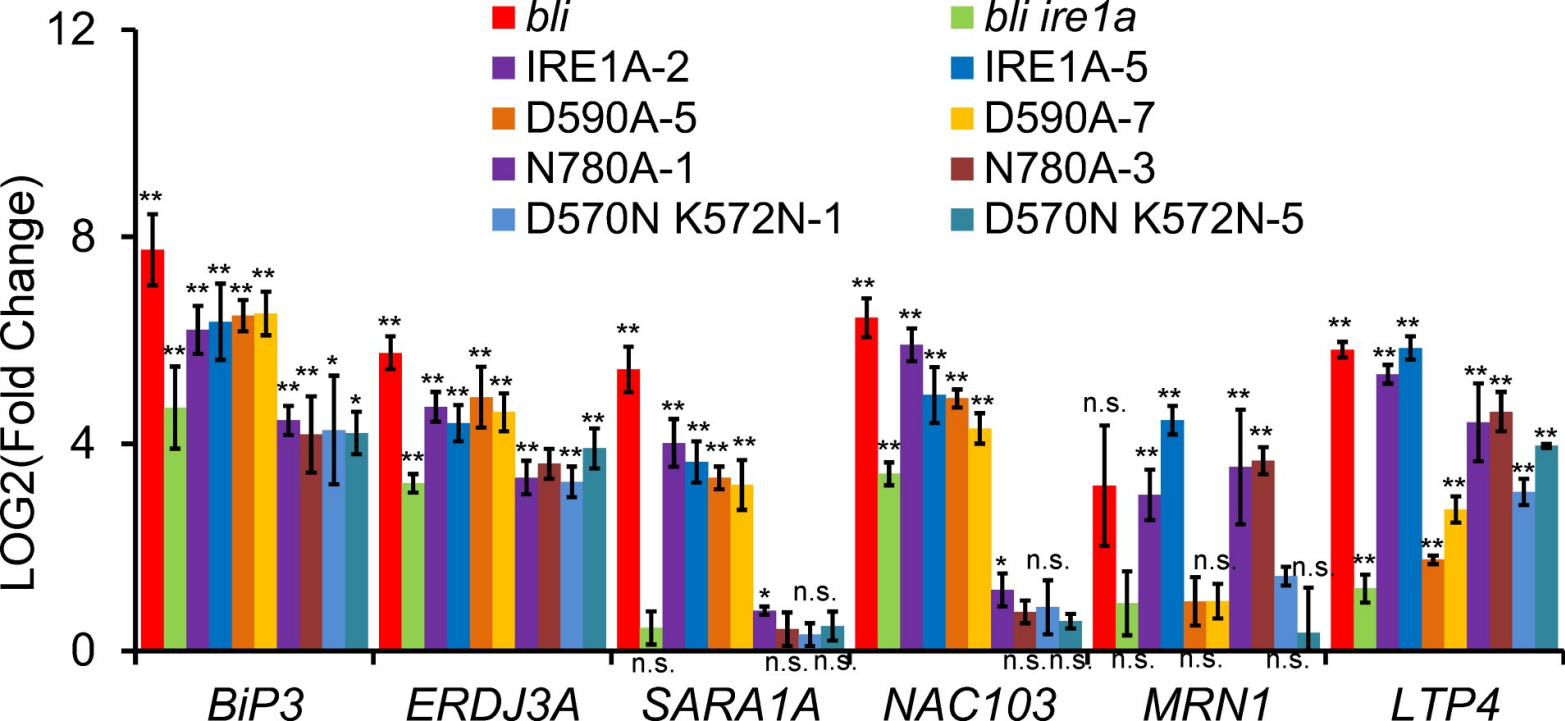
Residues in the protein kinase domain of IRE1A is required for non-canonical UPR gene expression in the BLI mutant background. T-DNA mutant *bli* (*bli-1*) was crossed to either *IRE1A* mutant (*ire1a*) or *IRE1B* mutant (*ire1b*) to generate the respective double mutant plants. The wild-type form and various mutated forms of IRE1A were expressed in the *bli ire1a* double mutant background and 2-weeks-old T2 transgenic plants were collected for gene expression analysis. Fold change is the gene expression level in the mutants or the genetically complemented materials normalized to that in the WT, both of which were normalized to the expression of *ACTIN*. Error bars represent SE (n = 3). Asterisks indicate significance levels when comparing to the WT control in *t*-test. (*, p<0.05; **, p<0.01; n.s., not significant at p<0.05).

It is known that IRE1B has a role in vegetative growth [30], we focused more on characterization of IRE1A in the current paper. Previously, it was shown that in yeast, D797N K799N (called 1KR32) mutations in the nucleotide-binding pocket abolish autophosphorylation and transautophosphorylation in *in vitro* kinase assays but retain the RNase activity in *in vitro* RNase assays [31]. D828A mutation in yeast abolishes autophosphorylation in *in vitro* kinase assays, although it retains ATP binding activity [32]. N1057A mutation of yeast IRE1 in the RNase domain only abolished its RNase activity [33]. We made equivalent mutations of IRE1A (Fig 5A and S6 Fig) and analyzed the autophosphorylation of IRE1A *in vitro*. Calf intestinal alkaline phosphatase (CIAP) is widely used to eliminate the phosphate group from phosphorylated proteins, and molecular weight shift after CIAP treatment has been successfully used for detection the phosphorylation status of mammalian IRE1 [34]. We have taken this advantage and detected IRE1A autophosphorylation on Phosphate-affinity polyacrylamide gel (Phos-tag) electrophoresis following CIAP treatment (Fig 5B). D590A (corresponding to yeast D828A) and D570N K572N (corresponding to yeast D797N K799N) mutations abolished the autophosphorylation of IRE1A, while N780A (corresponding to yeast N1057A) did not (Fig 5B). We then expressed these mutated forms of IRE1A or the non-mutated form of IRE1A driven by the *IRE1A* native promoter in the *bli ire1a* double mutant background (S7 Fig). Phenotypic analysis showed that expression of the non-mutated form of IRE1A (IRE1A) and the RNase dead form of IRE1A (N780A) reverted the *bli ire1a* phenotype to the *bli* phenotype (Fig 5C and 5D). However, expression of the kinase dead forms of IRE1A (D590A and D570N K572N) largely resembled the *bli ire1a* phenotype (Fig 5C and 5D). These results demonstrate that the protein kinase domain but not the RNase domain of IRE1A is required for BLI-regulated shoot growth phenotype. Further gene expression analysis showed that the up-regulation of canonical UPR marker genes was restored when IRE1A and D590A were expressed (Fig 6). In contrast, the up-regulation of UPR marker genes was not restored when N780A and D570N K572N were expressed (Fig 6). However, the up-regulation of non-canonical UPR marker genes (*MRN1* and *LTP4*) was restored when IRE1A and N780A were expressed (Fig 4D). Thus, the protein kinase domain of IRE1A is required for the *bli* mutant phenotype and up-regulation of non-canonical UPR genes such as *MRN1* in the *bli* mutant. D570N K572N mutation in the ATP-binding pocket and N780A mutation in the RNase domain affect IRE1A’s RNase activity to induce canonical UPR gene expression.

## Discussion

UPR signaling has protective functions in cells during ER stress response by increasing the ER protein folding capacity in the cell, enhancing the protein degradation of unfolded/misfolded proteins, and probably reducing the synthesis of new proteins in ER in plants. The Inositol-requiring enzyme 1 (IRE1) is one of the most important ER transmembrane sensors that activates the UPR for protein homeostasis under ER stress conditions through its specific splicing RNA target or non-specific RNA substrates in a process called RIDD [35]. However, IRE1’s activity needs to be timely attenuated after ER stress, and even under normal physiological conditions, IRE1’s activity needs to be finely tuned. For example, mutation of the transcription factor *SPL6* resulted in increased expression level of *IRE1* therefore elevated level of IRE1 protein, leading to constitutive ER stress response and developmental arrests in rice panicles [24]. However, in the current study, neither the expression of *IRE1A* nor *IRE1B* is affected in the *bli* mutant. We could not exclude the possibility that the protein level of IRE1A/IRE1B is affected in the *bli* mutant, since generation of antibody against *Arabidopsis* IRE1A/IRE1B is not successful and the rice IRE1 antibody [24] did not recognize the *Arabidopsis* IRE1A/IRE1B. However, our genetic complementation experiments demonstrated that the *bli* phenotype at seedling stage is dependent on the kinase domain of IRE1A, suggesting that the protein activity rather than the protein level of IRE1A is more probably affected by BLI. Little is understood on how IRE1’ activity is regulated in plants. In mammalian cells, it was proposed that, under normal conditions, the ER chaperone BiP/Grp78 binds to IRE1α to keep the protein in an inactive monomeric state, under ER-stressed conditions, releasing of BiP from IRE1α allows monomerization and autophosphorylation of its cytosolic domain, which triggers the activation of the RNase activity and downstream events [36]. However, whether similar mechanisms are existed in plants to tune the IRE1’s activity has not yet been reported. In the current study, we found that BLI is a negative regulator of IRE1 in Arabidopsis. Mutation of *BLI* results in activation of IRE1 and bZIP60 splicing, leading to increased expression level of canonical UPR genes such as *BiP3* and non-canonical UPR genes such as *MRN1* in plants. It seems that high level of BiP in *bli* mutant does not inhibit but activate IRE1A/IRE1B in *Arabidopsis*. Both *Arabidopsis* IRE1A and IRE1B were observed in perinuclear ER in tobacco BY2 cells [37]. Recently, IRE1B was also shown to be distributed in ER in Arabidopsis protoplasts under normal growth condition [38]. Given that BLI was observed in the nucleus and Golgi in *Arabidopsis* root cells, we hypothesized that BLI may indirectly control the activity of IRE1A/IRE1B. Further studies are needed to understand how BLI regulates IRE1’s activity in plants. Suppressor screening of *bli* mutant is underway in the lab.

The N-terminal part of IRE1 is in the ER lumen while the C-terminus is facing the cytoplasm which contains a protein kinase domain and an RNase domain. Previously, D608N K610N mutations of *Arabidopsis* IRE1B (corresponding to D797N K799N of yeast IRE1, 1KR32) in the nucleotide-binding pocket abolished IRE1B’s autophosphorylation and reduced its RNase activity to splice *bZIP60* mRNA, while the D628A mutation (corresponding to D828A mutation of yeast IRE1) within the conserved DFG kinase motif only abolished IRE1B’s autophosphorylation; the N820A mutation (corresponding to N1057A mutation of yeast IRE1) in the RNase domain only abolished IRE1B’s RNase activity in *Arabidopsis* during ER stress response [30]. However, how these conserved amino acids are involved in IRE1A’s activity is not reported. In the current study, we carried out experiments to characterize these important residues of IRE1A in Arabidopsis *bli* mutant background under normal growth conditions. Our results showed that D570A K572A mutations and N780A mutation of IRE1A reduced its ribonuclease activity for canonical UPR downstream gene expression while D570A K572A mutations and D590A mutation in the protein kinase domain of IRE1A affected its protein kinase activity for non-canonical UPR gene expression. Thus, the regulatory effect of protein kinase activity on its RNase activity is common for these two IRE1 proteins in Arabidopsis plants, which is different from yeast cells in which 1KR32 mutation in the nucleotide-binding pocket retains the RNase activity [32]. Autophosphorylation of IRE1B [30] and IRE1A (this study) has been demonstrated *in vitro*, however, detection of the autophosphorylation of these two proteins *in vivo* has not yet been successful, most probably because of the low level of these proteins in plants. Given that D590A mutation in IRE1A and D628A mutation in IRE1B do not affect the RNase activity of IRE1 in Arabidopsis, these results suggest that the ATP binding site of IRE1 rather than the protein kinase activity is more important for the activation of RNase activity in plants.

Besides the important roles of IRE1 in plant ER stress responses, the root-specific phenotype of Arabidopsis *ire1a ire1b* double mutant plants under unstressed conditions suggests that IRE1 also have other function in integrating physiological signals to maintain specific secretory activity [39]. Mutant analysis showed that the root growth phenotype of *ire1a ire1b* double mutant in the absence of stress was dependent on IRE1B [30]. Our results revealed further that the shoot growth retardation phenotype in *bli* mutant under normal growth conditions were dependent on IRE1A by analyzing the advance mutants in the *bli* mutant background. The root growth phenotype and reproductive development defects in *bli* mutant were not rescued by *IRE1A* or *IRE1B* mutation, suggesting that BLI also has IRE1-independent roles in plant growth and development, or IRE1A and IRE1B are too important for reproductive development so that the *bli ire1a* and *bli ire1b* double mutants could not recover. Nevertheless, our data demonstrated that the function of IRE1A in shoot growth regulation in *bli* mutant dependents on neither its RNase activity, nor its splicing target *bZIP60*. The protein kinase domain of IRE1A is required for the regulation of growth and development in *bli* mutant plants, probably through regulating of non-canonical UPR downstream genes.

**Fig 7:**
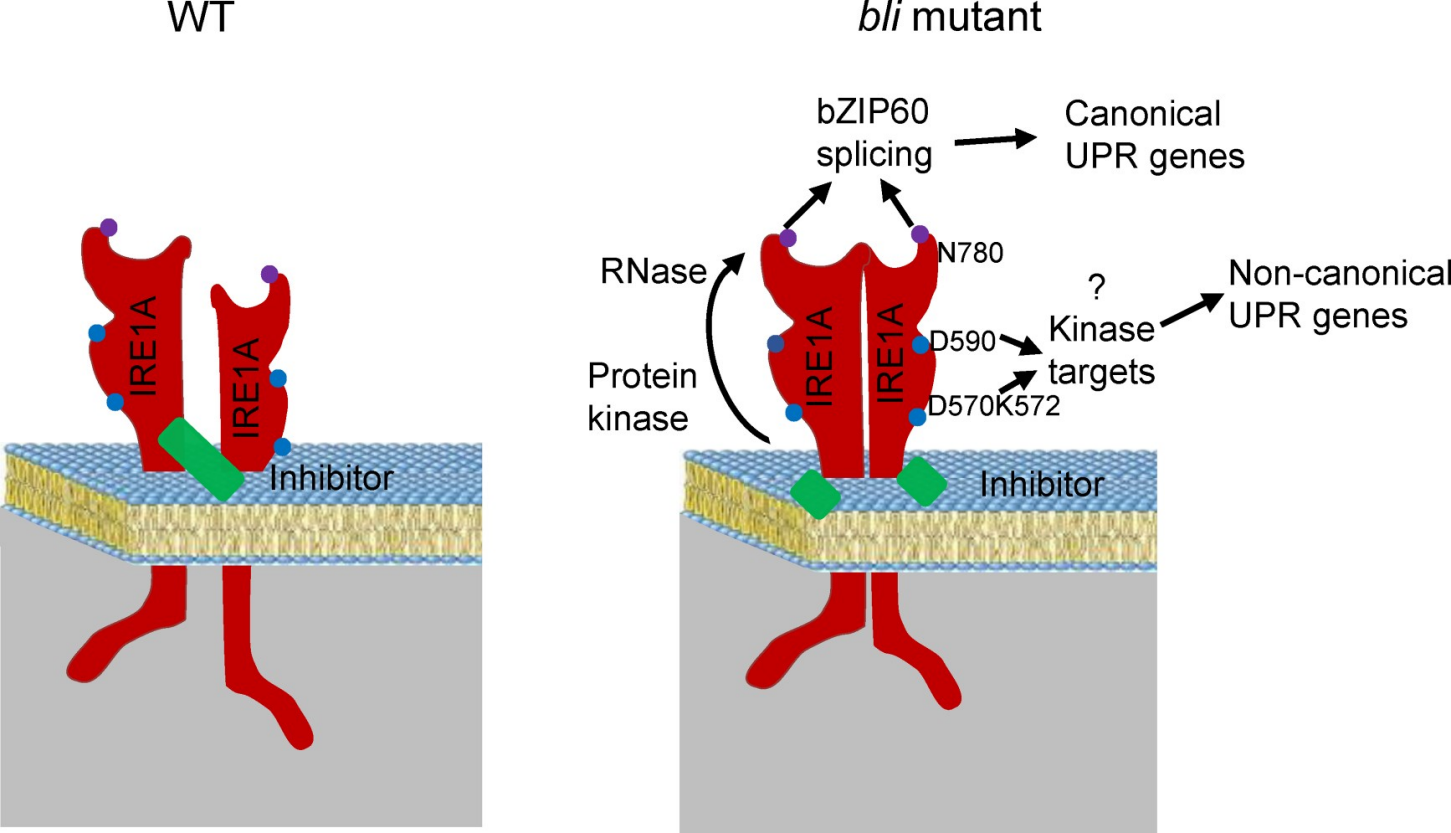
A working model for IRE1-mediated growth regulation in *BLI* mutant plants. In the presence of BLISTER (BLI) in wildtype (WT) plants, BLI may directly or indirectly inhibit IRE1A activation and clustering. In the absence of BLI in *bli* mutant plants, the inhibitor is removed and IRE1A is activated. The RNase activity of IRE1A for *bZIP60* mRNA splicing and downstream canonical UPR gene expression is dependent on N780 in the RNase domain and D570/K572 in the ATP-binding pocket of IRE1A. In contrast, the auto-phosphorylation activity of IRE1A is dependent on D570/K572 and D590 of IRE1A, which is important for non-canonical UPR gene expression and the shoot growth retardation phenotype.

In summary, we identified BLI as a negative regulator of IRE1A, constraining its function under normal growth conditions in *Arabidopsis* (Fig 7). In our working model, BLI may directly inhibit IRE1A’s function, or regulate the expression of an unknown protein to inhibit IRE1A’s function during vegetative growth. Once *BLI* is mutated, the activated IRE1A induces UPR genes and other non-canonical ER stress genes, leading to cell death and growth retardation in plants. D570/K572 in the ATP-binding pocket and N780 in the RNase domain are important for IRE1A to regulate canonical UPR genes while D570/K572 and D590 are important for its auto-phosphorylation and non-canonical UPR gene expression.

## Materials and methods

### Plant material and growth conditions

All of the *Arabidopsis thaliana* seeds used in this study are in the Columbia (Col) background. Seeds of WT, *bli-1* (SAIL_107_D04), *bli-11* (GABI-Kat_663H12), *bzip28* (SALK_132285), *bzip60* (SALK_050203), *ire1a* (SALK_018112), *ire1b* (SAIL_238_F07), double mutants and transgenic plants were sterilized (10 min 70% Ethanol supplemented with 0.05% Triton X-100, 10 min 96% Ethanol) and sown on germination medium (MS; half-strength Murashige and Skoog medium supplemented with 1% sucrose, 0.05% MES, and 0.8% plant agar). Seeds were stratified for two days at 4°C and grown under long day (LD) conditions, (16/8 h light / dark cycle at 22°C) in the medium or in the soil. For ER stress treatment, plants were grown on MS medium for 6 days and then transferred to either MS medium or MS plus 0.3 μg/ml tunicamycin (TM, Sigma) for additional 6 days. For silique length measurement, two longest siliques from each of the five main inflorescences were selected and measured for each genotype. For statistical analysis of gene expression, student’s t test was used. For multiple comparisons of phenotypes, LSD (Least Significant Difference) test was carried out following the ANOVA (Analysis of Variance) analysis.

### Trypan blue staining and DAB staining

Trypan blue staining and DAB staining were performed as described in previous literature [40]. For trypan blue staining, 14-day-old leaves of wild-type, *bli*, *bli ire1a* and *bli ire1b* were boiled for 1 min in trypan blue staining buffer containing 12.5% phenol, 12.5% glycerol, 12.5% lactic acid, 48% ethanol, and 0.025% trypan blue (Sangon Biotech), incubated for 10 min at room temperature, followed by destaining five times in 70% chloral hydrate. For DAB staining, leaves of 14-day-old wild-type, *bli*, *bli ire1a* and *bli ire1b* leaves were incubated in 1 mg/ mL DAB (Sigma) solution (pH 3.8) for 5 h at room temperature in darkness and then boiled in 95% boiling ethanol for 10 min before photograph.

### Genetic complementation

The IRE1A genomic sequences including 2.5 kb upstream promoter region were amplified from wild-type and inserted into pCambia 1300 to obtain the *IRE1A*:*IRE1A* construct. The site-specific-mutated PCR products were amplified by overlapping PCR based on the *IRE1A*:*IRE1A* construct. All the error-free constructs were introduced into the *bli+/- ire1a-/-* double mutant plants via Agrobacterium-mediated transformation. The T1 seeds were grown on selective MS medium plus 30 mg/L hygromycin (Roche) and genotyped to select transgenic plants in *bli-/- ire1a-/-* background. The T3 seeds were obtained and grown on MS medium for phenotypic analysis along with genotyping and gene expression analysis. At least five independent lines of each construct were selected and examined. All the primers used are listed in S1 Table.

### RNA-Seq and qRT-PCR

For RNA-Seq analyses, 14-days-old seeding were collected and immediately frozen in liquid nitrogen. Total RNA were extracted with Trizol (Invitrogen) and sequenced on the Illumina HiSeq 4000 platform by commercial company (Major Bio) following the standard Illumina protocols [41]. RNA-Seq reads were aligned to the reference genome of Arabidopsis thaliana (version TAIR10) using TopHat (version 2.0.13) (Kim et al., 2013) after filtering out low-quality (lowest base score < 20) reads using SeqPrep (https://github.com/jstjohn/SeqPrep) and Sickle (https://github.com/najoshi/sickle). Totally ~5.5–10 GB clean reads for each sample were obtained. Differential gene expression was assessed using Cuffdiff (http://cole-trapnell-lab.github.io/cufflinks/cuffdiff/index.html). The cutoff for significant differential expression was set as fold change (FC) ≥2 or FC≤0.5 and p-value p≤0.05. Parameters for sequencing quality control are listed in S2 Table. For qRT-PCR, RNA from 14-day-old seedlings were extracted using an RNA Prep Pure Plant kit (Tiangen). For reverse transcription, 2 mg of RNA and oligo (dT) primers were used to synthesize cDNA in a 20 μL reaction using M-MLV reverse transcriptase (TaKaRa). qRT-PCR was performed using SuperReal PreMix Color (Tiangen) in a CFX96 real-time system (Bio-Rad). There were three biological replicates for the RNA-Seq analysis. All the primers used are listed in S1 Table.

### Subcellular localization

Genomic *BLI*, containing the *BLI* coding region and 1.7 kb upstream of the transcriptional start site, was amplified from genomic DNA, and cloned into pGKGWG. The *BLI*:*BLI-GFP* construct was introduced to the *bli-11* mutant background and stable transformed plants were obtained. Confocal laser scanning microscopy was performed with root tissues using LSM 780 and LSM 510 microscopes (Zeiss). Image acquisition was carried out sequentially to prevent crosstalk between channels. GFP was excited at 488 nm, and emission was detected at 510–550 nm. RFP was excited at 561 nm and emission was detected at 575–630 nm. All the primers used are listed in S1 Table.

### De-phosphorylation assay

For de-phosphorylation assay, partial IRE1A sequences (AA345-841) were amplified from the wild-type Arabidopsis cDNA and cloned into pET28 to express the His-tag IRE1A truncation protein. Various mutated forms of IRE1A were amplified by overlapping PCR based on the wild-type form. All purified proteins (2 μg) were treated with or without CIAP (Takara) for 2 min at 37°C. The reaction were stopped by adding 4 μL 5X SDS buffer and boiling at 95°C for 5 min. All the proteins were resolved in Phos-tag gels and then immunoblotted with *anti*-His antibody (Abmart). All the primers used are listed in S1 Table.

### Accession numbers

Sequence data from this article can be found in the Arabidopsis Genome Initiative under the following accession numbers: At3g10800 (*bZIP28*), AT1G42990 (*bZIP60*), AT2G17520 (*IRE1A*), AT5G24360 (*IRE1B*), AT3G23980 (*BLI*), AT1G09080 (*BiP3*), AT4G21730 (*NSF*), AT3G08970, (*ERDJ3A*), AT3G62600, (*ERDJ3B*), AT1G09180 (*SARA1*), AT5G64060 (*NAC103*), AT5G42600 (*MRN1*) and AT5G59310 (*LTP4*). The RNA-Seq data in this article can be found in the Gene Expression Omnibus (GEO) under the accession number GSE124235.

## Supporting information

S1 Fig Mutation of *BLI* induces UPR gene expression. **A**, UPR gene expression analysis. Total RNA was exacted from 2-week-old plants for qRT-PCR. Fold change is the gene expression level in the *BLI* mutants (*bli-1* or *bli-11*) normalized to that in the wild-type plants (WT), both of which were normalized to the expression of *ACTIN*. Error bars represent SE (n = 3). *bZIP60U*, unspliced *bZIP60*; *bZIP60S*, spliced *bZIP60*. Asterisks indicate significance levels when comparing to the WT control in *t*-test. (*, p<0.05; **, p<0.01). **B**, Venn diagrams showing the overlapping regulated genes between *BLI*-dependent genes and canonical UPR genes. Canonical UPR genes were obtained by comparing gene expression profiles of WT plants treated with or without ER stress inducer tunicamycin (5 μg/ml) for 12 hours followed by RNA-Seq analysis. Up-regulation: fold change ≥2, p<0.05; down-regulation: fold change ≤0.5, p<0.05.
(PDF)

S2 Fig Mutation of either *IRE1A*, *IRE1B*, *bZIP28*, or *bZIP60* alone does not affect vegetative growth and reproductive development. **A-B**, Phenotypic analysis. T-DNA mutants of *IRE1A* (*ire1a*), or *IRE1B* (*ire1b*) or *bZIP28* (*bzip28*) or *bZIP60* (*bzip60*) were grown together with wild-type plants (WT) in standard MS growth medium. 2-week-old plant seedlings were photographed (A) and siliques lengths were measured at reproductive stage (B). Error bars represent SD (n = 10). Letters above the bars indicate significant differences as determined by LSD test following ANOVA analysis (p<0.05). Bar = 5 mm. **C**, UPR gene expression analysis. Total RNA was exacted from 2-week-old plants for qRT-PCR analysis. Error bars represent SE (n = 3). Asterisks indicate significance levels when comparing to the WT control in *t*-test. (*, p<0.05; **, p<0.01). *bZIP60U*, unspliced *bZIP60*; *bZIP60S*, spliced *bZIP60*.
(PDF)

S3 Fig Characterization of T-DNA mutants. Total RNA was extracted from various plant materials grown under normal growth conditions and the expression of *BLI*, *IRE1A* or *IRE1B* was checked by RT-PCR. *UBQ5* was used as a loading control.
(PDF)

S4 Fig The *BLI* ER stress related phenotype is partially suppressed by *IRE1A* or *IRE1B* mutation. (A-B) ER stress related phenotype of mutant plants. T-DNA mutant of *BLI* (*bli-1*) was crossed to either *IRE1A* mutant (*ire1a*) or *IRE1B* mutant (*ire1b*) to generate the respective double mutant plants. Wild-type (WT), single mutants and double mutants were vertically grown for 6 days on MS medium, transferred to either MS or MS plus 0.3 μg/ml tunicamycin (TM) plates and grown for additional 6 days, then photographed (A). Primary root length was measured (B). There were five plants in each of the three replicates. Error bars represent SE (n = 3). Letters above the bars indicate significant differences as determined by LSD test following ANOVA analysis (p<0.05). Bar = 10 mm.
(PDF)

S5 Fig Analysis of siliques from self-crossing plants. WT and *ire1a ire1b bli+/-* plants were grown in soils in well-controlled growth chamber and their siliques were examined after self-crossing. Arrow heads point to the aborted embryos.
(PDF)

S6 Fig Multiple sequence alignment of the IRE1’ C-termini. Protein sequences of the Arabidopsis IRE1s (AtIRE1A, At2g17520; AtIRE1B, At5g24360), rice IRE1 (OsIRE1, LOC_Os07g28820), yeast IRE1 (ScIRE1, NP_011946) and human IRE1 (HsIRE1, NP_001424) were subjected to sequence alignment using CLUSTAL 2.1 with the default setting. Only the C-terminal regions beyond the transmembrane domains are shown. The protein kinase domain is shaded in grey while the endonuclease domain is shaded in yellow. Amino acids predicted to be important for the protein kinase activity or endonuclease activity are highlighted in red.
(PDF)

S7 Fig Validation of transgene expression. The wild-type form and various mutated forms of IRE1A were expressed the *bli ire1a* double mutant background. Total RNA was extracted from various plant materials grown under normal growth conditions and the expression of total *IRE1A* was checked by RT-PCR. *UBQ5* was used as a loading control.
(PDF)

S1 Table Primers used in this study. (PDF)

S2 Table Parameters for sequencing quality control of each sample. (PDF)

S1 Dataset Differentially regulated genes. (XLSX)
