# Supplementary material for: BLISTER-regulated vegetative growth is dependent on the protein kinase domain of ER stress modulator IRE1A in Arabidopsis thaliana
Source: PLoS Genet. 2019 Dec 23;15(12):e1008563. doi: 10.1371/journal.pgen.1008563 (PMC6946172; doi:10.1371/journal.pgen.1008563)
Supplement: S1 Fig — A, UPR gene expression analysis. Total RNA was exacted from 2-week-old plants for qRT-PCR. Fold change is the gene expression level in the BLI mutants (bli-1 or bli-11) normalized to that in the wild-type plants (WT), both of which were normalized to the expression of ACTIN. Error bars represent SE (n = 3). bZIP60U, unspliced bZIP60; bZIP60S, spliced bZIP60. Asterisks indicate significance levels when comparing to the WT control in t-test. (*, p<0.05; **, p<0.01). B, Venn diagrams showing the overlapping regulated genes between BLI-dependent genes and canonical UPR genes. Canonical UPR genes were obtained by comparing gene expression profiles of WT plants treated with or without ER stress inducer tunicamycin (5 μg/ml) for 12 hours followed by RNA-Seq analysis. Up-regulation: fold change ≥2, p<0.05; down-regulation: fold change ≤0.5, p<0.05. (PDF) [file pgen.1008563.s001.pdf]

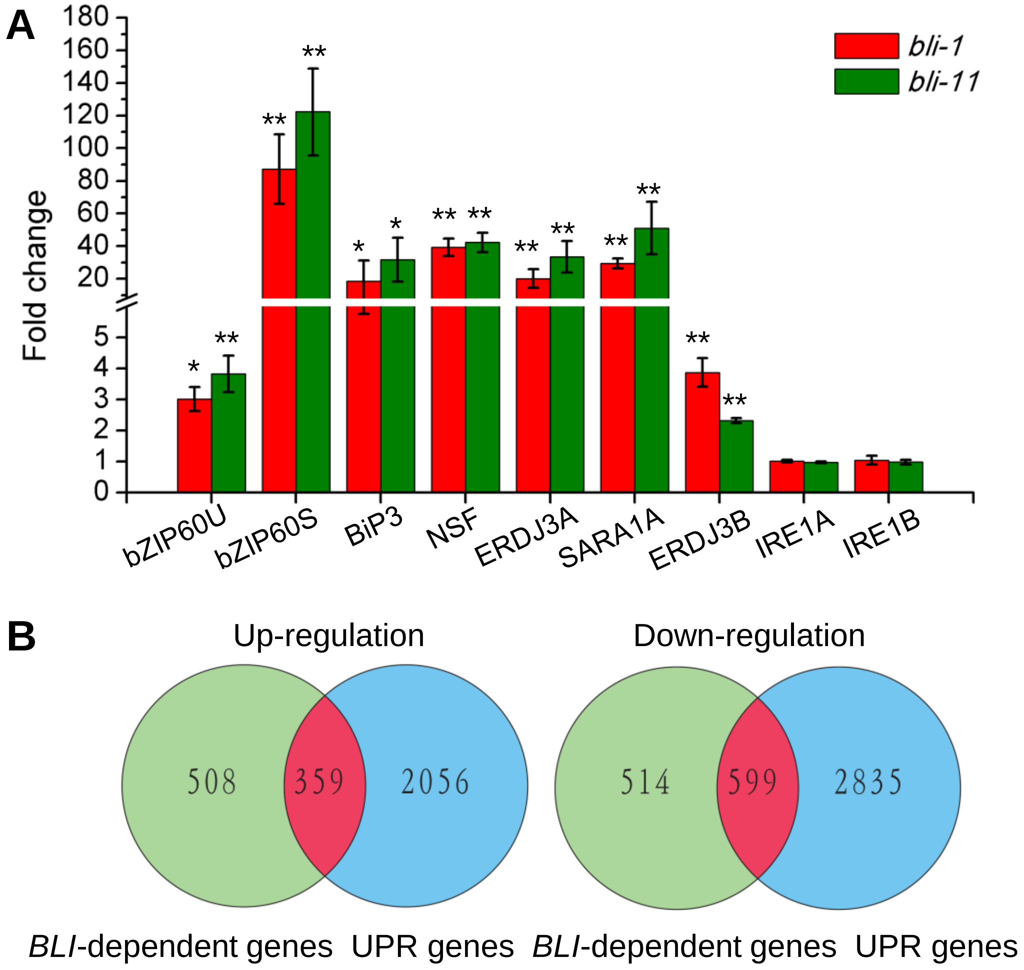

Fig S1. Mutation of *BLI* induces UPR gene expression.

A, UPR gene expression analysis. Total RNA was exacted from 2-week-old plants for qRT-PCR. Fold change is the gene expression level in the *BLI* mutants (*bli-1* or *bli-11*) normalized to that in the wild-type plants (WT), both of which were normalized to the expression of *ACTIN*. Error bars represent SE (n=3). *bZIP60U*, unspliced *bZIP60*; *bZIP60S*, spliced *bZIP60*. Asterisks indicate significance levels when comparing to the WT control in *t*-test. (*, p<0.05; **, p<0.01). **B**, Venn diagrams showing the overlapping regulated genes between *BLI*-dependent genes and canonical UPR genes. Canonical UPR genes were obtained by comparing gene expression profiles of WT plants treated with or without ER stress inducer tunicamycin (5 μg/ml) for 12 hours followed by RNA-Seq analysis. Up-regulation: fold change ≥2, p＜0.05; down-regulation: fold change ≤0.5, p＜0.05.
