# Supplementary material for: BLISTER-regulated vegetative growth is dependent on the protein kinase domain of ER stress modulator IRE1A in Arabidopsis thaliana
Source: PLoS Genet. 2019 Dec 23;15(12):e1008563. doi: 10.1371/journal.pgen.1008563 (PMC6946172; doi:10.1371/journal.pgen.1008563)
Supplement: S2 Fig — A-B, Phenotypic analysis. T-DNA mutants of IRE1A (ire1a), or IRE1B (ire1b) or bZIP28 (bzip28) or bZIP60 (bzip60) were grown together with wild-type plants (WT) in standard MS growth medium. 2-week-old plant seedlings were photographed (A) and siliques lengths were measured at reproductive stage (B). Error bars represent SD (n = 10). Letters above the bars indicate significant differences as determined by LSD test following ANOVA analysis (p<0.05). Bar = 5 mm. C, UPR gene expression analysis. Total RNA was exacted from 2-week-old plants for qRT-PCR analysis. Error bars represent SE (n = 3). Asterisks indicate significance levels when comparing to the WT control in t-test. (*, p<0.05; **, p<0.01). bZIP60U, unspliced bZIP60; bZIP60S, spliced bZIP60. (PDF) [file pgen.1008563.s002.pdf]

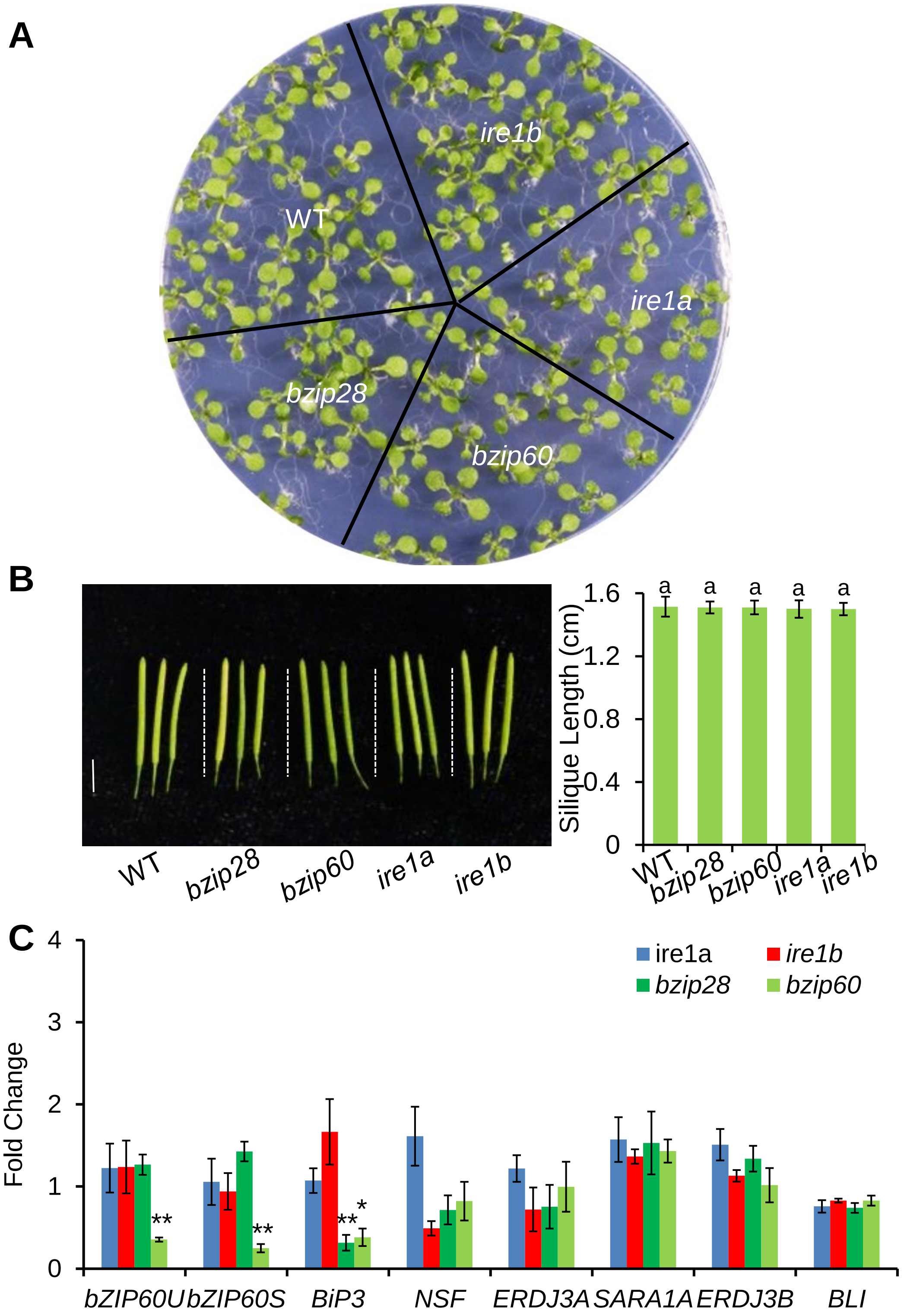

Fig S2. Mutation of either *IRE1A, IRE1B, bZIP28,* or *bZIP60* alone does not affect vegetative growth and reproductive development.

A-B, Phenotypic analysis. T-DNA mutants of *IRE1A* (*ire1a*), or *IRE1B* (*ire1b*) or *bZIP28* (*bzip28*) or *bZIP60* (*bzip60*) were grown together with wild-type plants (WT) in standard MS growth medium. 2-week-old plant seedlings were photographed (A) and siliques lengths were measured at reproductive stage (B). Error bars represent SD (n=10). Letters above the bars indicate significant differences as determined by LSD test following ANOVA analysis ($p<0.05$). Bar=5 mm. **C**, UPR gene expression analysis. Total RNA was exacted from 2-week-old plants for qRT-PCR analysis. Error bars represent SE (n=3). Asterisks indicate significance levels when comparing to the WT control in *t*-test. (*, $p<0.05$; **, $p<0.01$). *bZIP60U*, unspliced *bZIP60*; *bZIP60S*, spliced *bZIP60*.
