# Supplementary material for: BLISTER-regulated vegetative growth is dependent on the protein kinase domain of ER stress modulator IRE1A in Arabidopsis thaliana
Source: PLoS Genet. 2019 Dec 23;15(12):e1008563. doi: 10.1371/journal.pgen.1008563 (PMC6946172; doi:10.1371/journal.pgen.1008563)
Supplement: S3 Fig — Total RNA was extracted from various plant materials grown under normal growth conditions and the expression of BLI, IRE1A or IRE1B was checked by RT-PCR. UBQ5 was used as a loading control. (PDF) [file pgen.1008563.s003.pdf]

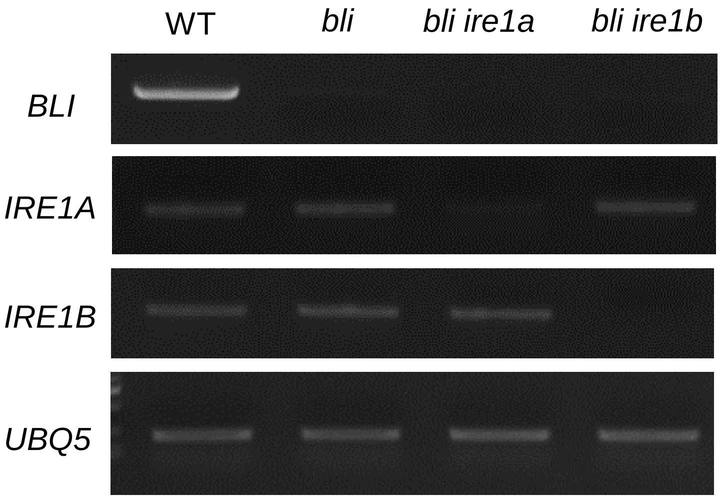

Fig S3. Characterization of T-DNA mutants.

Total RNA was extracted from various plant materials grown under normal growth conditions and the expression of *BLI*, *IRE1A* or *IRE1B* was checked by RT-PCR. *UBQ5* was used as a loading control.
