# Supplementary material for: BLISTER-regulated vegetative growth is dependent on the protein kinase domain of ER stress modulator IRE1A in Arabidopsis thaliana
Source: PLoS Genet. 2019 Dec 23;15(12):e1008563. doi: 10.1371/journal.pgen.1008563 (PMC6946172; doi:10.1371/journal.pgen.1008563)
Supplement: S4 Fig — (A-B) ER stress related phenotype of mutant plants. T-DNA mutant of BLI (bli-1) was crossed to either IRE1A mutant (ire1a) or IRE1B mutant (ire1b) to generate the respective double mutant plants. Wild-type (WT), single mutants and double mutants were vertically grown for 6 days on MS medium, transferred to either MS or MS plus 0.3 μg/ml tunicamycin (TM) plates and grown for additional 6 days, then photographed (A). Primary root length was measured (B). There were five plants in each of the three replicates. Error bars represent SE (n = 3). Letters above the bars indicate significant differences as determined by LSD test following ANOVA analysis (p<0.05). Bar = 10 mm. (PDF) [file pgen.1008563.s004.pdf]

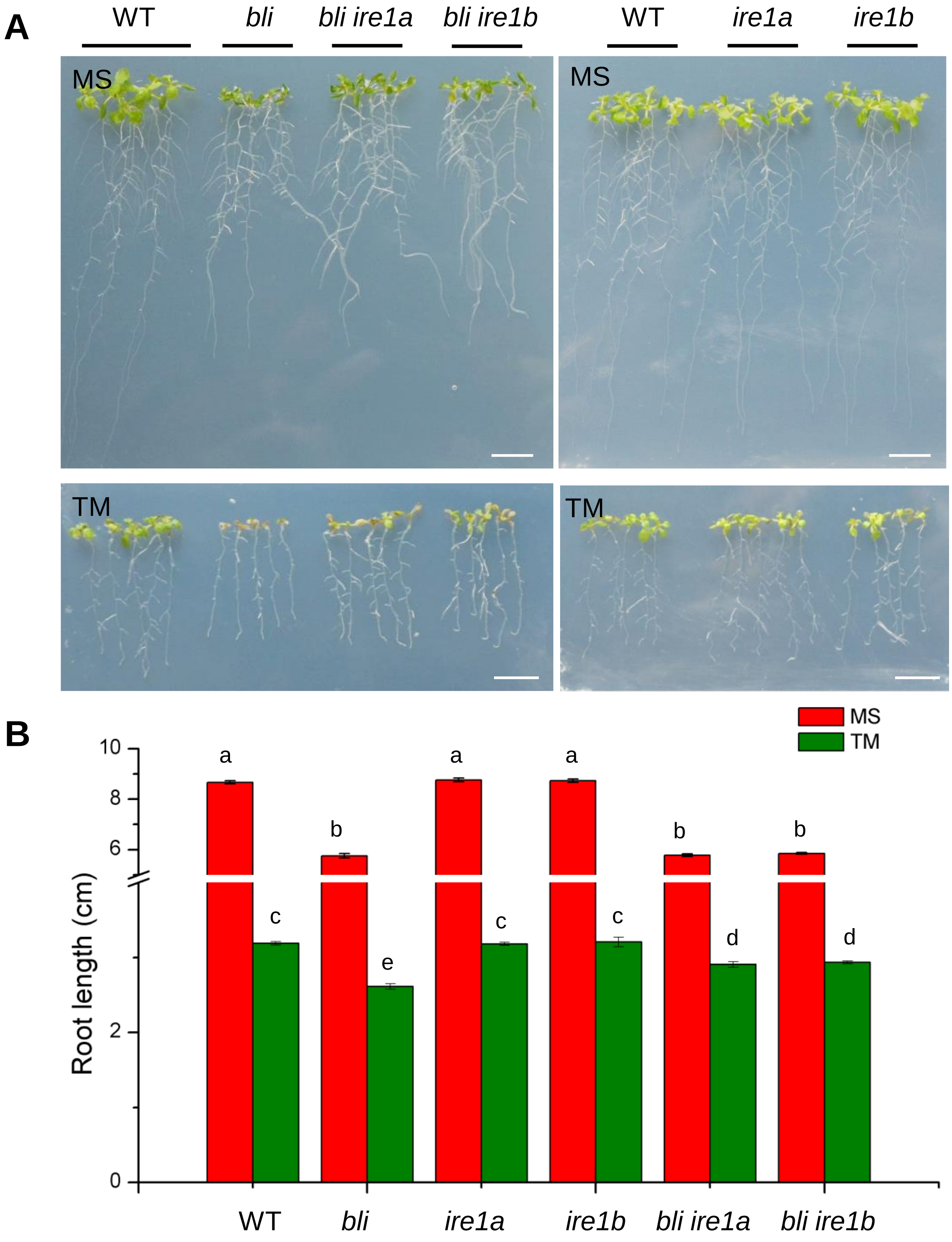

Fig S4. The *BLI* ER stress related phenotype is partially suppressed by *IRE1A* or *IRE1B* mutation.

(A-B) ER stress related phenotype of mutant plants. T-DNA mutant of *BLI* (*bli-1*) was crossed to either *IRE1A* mutant (*ire1a*) or *IRE1B* mutant (*ire1b*) to generate the respective double mutant plants. Wild-type (WT), single mutants and double mutants were vertically grown for 6 days on MS medium, transferred to either MS or MS plus 0.3 µg/ml tunicamycin (TM) plates and grown for additional 6 days, then photographed (A). Primary root length was measured (B). There were five plants in each of the three replicates. Error bars represent SE (n=3). Letters above the bars indicate significant differences as determined by LSD test following ANOVA analysis (p<0.05). Bar= 10 mm.
