# Supplementary material for: BLISTER-regulated vegetative growth is dependent on the protein kinase domain of ER stress modulator IRE1A in Arabidopsis thaliana
Source: PLoS Genet. 2019 Dec 23;15(12):e1008563. doi: 10.1371/journal.pgen.1008563 (PMC6946172; doi:10.1371/journal.pgen.1008563)
Supplement: S5 Fig — WT and ire1a ire1b bli+/- plants were grown in soils in well-controlled growth chamber and their siliques were examined after self-crossing. Arrow heads point to the aborted embryos. (PDF) [file pgen.1008563.s005.pdf]

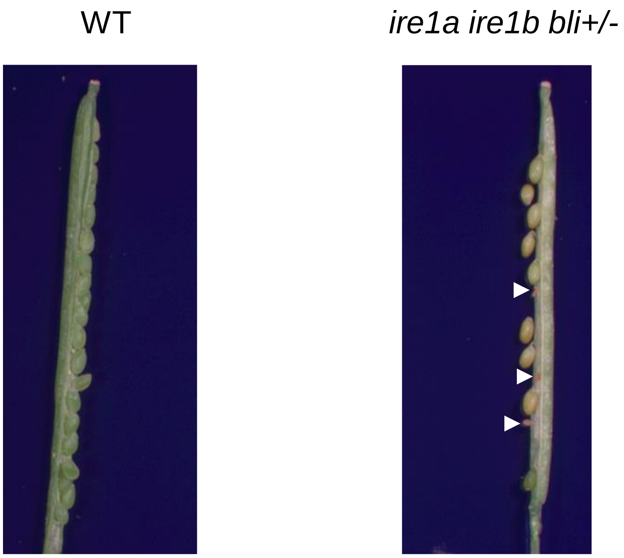


**Fig S5. Analysis of siliques from self-crossing plants.**

WT and *ire1a ire1b bli+/-* plants were grown in soils in well-controlled growth chamber and their siliques were examined after self-crossing. Arrow heads point to the aborted embryos.
