# Supplementary material for: BLISTER-regulated vegetative growth is dependent on the protein kinase domain of ER stress modulator IRE1A in Arabidopsis thaliana
Source: PLoS Genet. 2019 Dec 23;15(12):e1008563. doi: 10.1371/journal.pgen.1008563 (PMC6946172; doi:10.1371/journal.pgen.1008563)
Supplement: S6 Fig — Protein sequences of the Arabidopsis IRE1s (AtIRE1A, At2g17520; AtIRE1B, At5g24360), rice IRE1 (OsIRE1, LOC_Os07g28820), yeast IRE1 (ScIRE1, NP_011946) and human IRE1 (HsIRE1, NP_001424) were subjected to sequence alignment using CLUSTAL 2.1 with the default setting. Only the C-terminal regions beyond the transmembrane domains are shown. The protein kinase domain is shaded in grey while the endonuclease domain is shaded in yellow. Amino acids predicted to be important for the protein kinase activity or endonuclease activity are highlighted in red. (PDF) [file pgen.1008563.s006.pdf]

```
AtIRE1A     ---------IGKLFLSSK-EIAKGSNGTVVFEGIYEGRPVAVKRLVRSHHEVAFKEIQNL 459
AtIRE1B     ---------VGKLFVSNK-EIAKGSNGTVVLEGSYEGRLVAVKRLVQSHHDVAQKEILNL 504
OsIRE1      ---------IGKLCVYSK-EIGKGSNGTVVFEGSYGGREVAVKRLLRSHNDIASKEIENL 536
ScIRE1      ANIPNFEQSLKNLVVSEK-ILGYGSSGTVVFQGSFQGRPVAVKRMLIDFCDIALMEIKLL 719
HsIRE1      ----TSVVIVGKISFCPKDVLGHGAEGTIVYRGMFDNRDVAVKRILPECFSFADREVQLL 616
                     : :: .  *  :. *:.**:* .* : .* *****:: .  ..*  *:  *

AtIRE1A     IASDQHTNIIRWYGVEYDQDFVYLSLERCTCSLDDLIKSYLEFSMTKVLENNDSTEGVAA 519
AtIRE1B     MASDKHSNIVRWYGVDQDEHFIYISLELCACSLNDLI-----YASSALLESPMASS--SI 557
OsIRE1      IASDQDPNIVRMYGFEQDNDFVYISLERCRCSLADLIQ----LHSVPPFSNTKGTD---- 588
ScIRE1      TESDDHPNVIRYYCSETTDRFLYIALELCNLNLQDLVE-----------SKNVSDE---- 764
HsIRE1      RESDEHPNVIRYFCTEKDRQFQYIAIELCAATLQEYVE---------------------- 654
              **...*::* :  :    * *:::* *  .* : :

AtIRE1A     YKIQLDSLEGVIKGNNFWKVGGHPSPLMLKLMRDIVCGIVHLHELGIVHRDLKPQNVLIS 579
AtIRE1B     HSIQINPIFENGKGVELWKENGHPSPVLLKLMRDIVAGLVHLHDIGIVHRDLKPQNVLIV 617
OsIRE1      --------------IELWRQDGLPSAQLLKLMRDVVAGIVHLHSLGIIHRDLKPQNVLIS 634
ScIRE1      ------------------NLKLQKEYNPISLLRQIASGVAHLHSLKIIHRDLKPQNILVS 806
HsIRE1      -----------------QKDFAHLGLEPITLLQQTTSGLAHLHSLNIVHRDLKPHNILIS 697
                              .         :.*::: ..*:.***.: *:******:*:*:

AtIRE1A     KDM-----------TLSAKLSDMGISKRMSRDMSSLGHLAT--GSGSSGWQAPEQLLQG- 625
AtIRE1B     KNS-----------SLCAKLSDMGISKRLPADTSALTRNSTGLGSGSSGWQAPEQLRNE- 665
OsIRE1      KEG-----------PLRAKLSDMGISKRLQEDMTSVSHHGT--GFGSSGWQAPEQLRHG- 680
ScIRE1      TSSRFTADQQTGAENLRILISDFGLCKKLDSGQSSFRTNLNN-PSGTSGWRAPELLEESN 865
HsIRE1      MPNAHG--------KIKAMISDFGLCKKLAVGRHSFSRRSG--VPGTEGWIAPEMLSEDC 747
                           :   :**:*:.*::  .  :.         *:.** *** * .

AtIRE1A     ------------------------------RQTRAVDMFSLGCVIFYTITGCKHPFGDDL 655
AtIRE1B     ------------------------------RQTRAVDLFSLGCVLFFCMTGGKHPYGDNY 695
OsIRE1      ------------------------------RQTRAIDLFSLGCLIFYCITKGKHPFGEYY 710
ScIRE1      NLQCQVETEHSSSRHTVVSSDSFYDPFTKRRLTRSIDIFSMGCVFYYILSKGKHPFGDKY 925
HsIRE1      K----------------------------ENPTYTVDIFSAGCVFYYVISEGSHPFGKSL 779
                                          . * ::*:** **:::: ::  .**:*.

AtIRE1A     ERDVNIVK------NKVDLFLVEHVPEAS-DLISRLLNPDPDLRPSATEVLLHPMFWNSE 708
AtIRE1B     ERDVNVLN------DQKDLFLIESLPEAV-HLLTGLLNPDPNLRPRAQDVMHHPLFWNSD 748
OsIRE1      ERDMKIIN------NQFDLFIVDHIPEAV-HLISQLLDPDPEKRPTAVYVMHHPFFWSPE 763
ScIRE1      SRESNIIRGIFSLDEMKCLHDRSLIAEAT-DLISQMIDHDPLKRPTAMKVLRHPLFWPKS 984
HsIRE1      QRQANILLG---ACSLDCLHPEKHEDVIARELIEKMIAMDPQKRPSAKHVLKHPFFWSLE 836
            .*: :::       .   *.  .       .*:  ::  **  ** *  *: **:**  .

AtIRE1A     MRLSFLRDASDRVELENREADSEILKAMES-TAPVAIGGKWDEKLEPVFITNIGRYRRYK 767
AtIRE1B     MRLSFLRDASDRVELENREEGSQLLAALES-TAAVTLNGRWDEKLDSIFLDNIGRYRRYK 807
OsIRE1      LCLSFLRDTSDRIEKTS---ETDLIDALEG-INVEAFGKNWGEKLDAALLADMGRYRKYS 819
ScIRE1      KKLEFLLKVSDRLEIENRDPPSALLMKFDAGSDFVIPSGDWTVKFDKTFMDNLERYRKYH 1044
HsIRE1      KQLQFFQDVSDRIEKES--LDGPIVKQLER-GGRAVVKMDWRENITVPLQTDLRKFRTYK 893
              *.*: ..***:*  .      ::  ::           *  ::   :  ::  ::* *

AtIRE1A     YDSIRDLLRVIRNKLNHHRELPPEIQELVGTVPEGFDEYFAVRFPKLLIEVYRVISLHCR 827
AtIRE1B     FDSIRDLLRVIRNKLNHYRELPKELQELLGSVPEGFERYFSSRFPKLLIQVYTVLFDYCN 867
OsIRE1      FESTRDLLRLIRNKSGHYREFSDDLKELLGSLPEGFVQYFSSRFPKLLIKVYEVMSEHCK 879
ScIRE1      SSKLMDLLRALRNKYHHFMDLPEDIAELMGPVPDGFYDYFTKRFPNLLIGVYMIVKENLS 1104
HsIRE1      GGSVRDLLRAMRNKKHHYRELPAEVRETLGSLPDDFVCYFTSRFPHLLAHTYRAMELCSH 953
              .  **** :***  *. ::. :: * :*.:*:.*  **: ***:**  .*  :

AtIRE1A     EEEVFRKYFKCDII---------- 841
AtIRE1B     NEEFFFKYSKTTVF---------- 881
OsIRE1      DEEAFSKYFLGSSA---------- 893
ScIRE1      DDQILREFLYS------------- 1115
HsIRE1      ERLFQPYYFHEPPEPQPPVTPDAL 977
            :      :
```

Fig S6. Multiple sequence alignment of the IRE1' C-termini.

Protein sequences of the Arabidopsis IRE1s (AtIRE1A, At2g17520; AtIRE1B, At5g24360), rice IRE1 (OsIRE1, LOC_Os07g28820), yeast IRE1 (ScIRE1, NP_011946) and human IRE1 (HsIRE1, NP_001424) were subjected to sequence alignment using CLUSTAL 2.1 with the default setting. Only the C-terminal regions beyond the transmembrane domains are shown. The protein kinase domain is shaded in grey while the endonuclease domain is shaded in yellow. Amino acids predicted to be important for the protein kinase activity or endonuclease activity are highlighted in red.
