# Supplementary material for: BLISTER-regulated vegetative growth is dependent on the protein kinase domain of ER stress modulator IRE1A in Arabidopsis thaliana
Source: PLoS Genet. 2019 Dec 23;15(12):e1008563. doi: 10.1371/journal.pgen.1008563 (PMC6946172; doi:10.1371/journal.pgen.1008563)
Supplement: S7 Fig — The wild-type form and various mutated forms of IRE1A were expressed the bli ire1a double mutant background. Total RNA was extracted from various plant materials grown under normal growth conditions and the expression of total IRE1A was checked by RT-PCR. UBQ5 was used as a loading control. (PDF) [file pgen.1008563.s007.pdf]

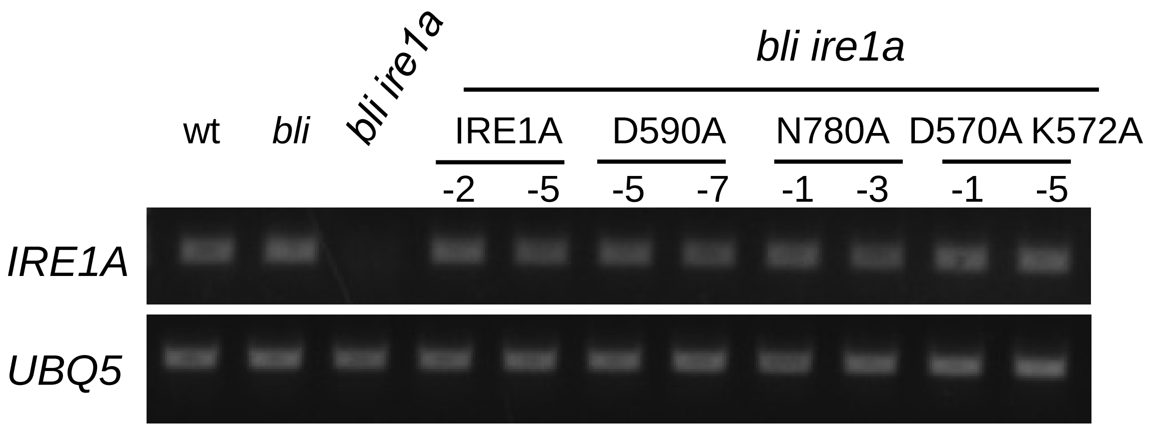

Fig S7. Validation of transgene expression.

The wild-type form and various mutated forms of IRE1A were expressed the *bli ire1a* double mutant background. Total RNA was extracted from various plant materials grown under normal growth conditions and the expression of total *IRE1A* was checked by RT-PCR. *UBQ5* was used as a loading control.
