# Supplementary material for: BLISTER-regulated vegetative growth is dependent on the protein kinase domain of ER stress modulator IRE1A in Arabidopsis thaliana
Source: PLoS Genet. 2019 Dec 23;15(12):e1008563. doi: 10.1371/journal.pgen.1008563 (PMC6946172; doi:10.1371/journal.pgen.1008563)
Supplement: S1 Table — (PDF) [file pgen.1008563.s008.pdf]

Table S1. Primers used in this study.

Name	Locus	Purpose	Primer sequences(5'-3')
qBiP3F	At1g09080	qRT-PCR	CACGGTTCCAGCGTATTTCAAT
qBiP3R	At1g09080	qRT-PCR	ATAAGCTATGGCAGCACCCGTT
qNSFF	At4g21730	qRT-PCR	CATGGCTGATTCTTCCTATT
qNSFR	At4g21730	qRT-PCR	AACTTCTCCGACGTTAGCGA
qERDJ3AF	At3g08970	qRT-PCR	GGTGGTGGTTTCAACTTTGG
qERDJ3AR	At3g08970	qRT-PCR	GAACTACCGCCCGAAAACAT
qERDJ3BF	At3g62600	qRT-PCR	TTTGGTGGAGGTTCGATGGA
qERDJ3BR	At3g62600	qRT-PCR	ACCTCGTTCCTGCAGTTACA
qSARA1F	At1g09180	qRT-PCR	GTTGATGCTGTTGTCTACCT
qSARA1R	At1g09180	qRT-PCR	CTTCTGAGGATGCATAAGGT
qNAC103F	At5g64060	qRT-PCR	AAGTCGTAGAGCTACAGGAT
qNAC103R	At5g64060	qRT-PCR	TAGAAACGTTCTGGCTAAAG
qMRN1F	At5g42600	qRT-PCR	TCTTGCTCGAGTCCCTGATT
qMRN1R	At5g42600	qRT-PCR	TGGTCACCTTGAGGGTTCTC
qLTP4F	At5g59310	qRT-PCR	CTATCAAAGGGTGGGGTGGT
qLTP4R	At5g59310	qRT-PCR	GTGCTCGTGGAGATGGGATA
qbZIP60UF	At1g42990	qRT-PCR	GAAGGAGACGATGATGCTGTGGCT
qbZIP60UR	At1g42990	qRT-PCR	GCAGGGATTCCAACAAGAGCACAG
qbZIP60SF	At1g42990	qRT-PCR	GAAGGAGACGATGATGCTGTGGCT
qbZIP60SR	At1g42990	qRT-PCR	AGCAGGGAACCCAACAGCAGACT
qACTINF	At3g18780	qRT-PCR	GGTAACATTGTGCTCAGTGGTGG
qACTINR	At3g18780	qRT-PCR	AACGACCTTAATCTTCATGCTGC
qIRE1AF	At2g17520	qRT-PCR	GCGCTACAGGCGTTACAAATA
qIRE1AR	At2g17520	qRT-PCR	TCGTCGAATCCTTCTGGAACT
qIRE1BF	At5g24360	qRT-PCR	ACAACTCCGCAATGAACGAC
qIRE1BR	At5g24360	qRT-PCR	GGGTTTAGGAGACCAGTGAGAA
qBLIF	At3g23980	qRT-PCR	GCTTGTAACAGCGCAGAAGA
qBLIR	At3g23980	qRT-PCR	TTCTTTTCGACGGTCCTCCT
IRE1AF	At2g17520	RT-PCR	GCGCTACAGGCGTTACAAATA
IRE1AR	At2g17520	RT-PCR	TCGTCGAATCCTTCTGGAACT
UBQ5F	At3g62250	RT-PCR	TTGAAGACGGCCGTACCCTC
UBQ5R	At3g62250	RT-PCR	CGCTGAACCTTTCAAGATCCATCG
pro-IRE1A-F	At2g17520	Promoter cloning	GCGAGCTCGCAATTCAGGTTGTCCAAGT
pro-IRE1A-R	At2g17520	Promoter cloning	GCGTCGACAAACCCCCACGAACGAACCA
gIRE1A-F	At2g17520	Complementation	CCGCTCGAGGGAGAACGCGTTAGACACAC
gIRE1A-R	At2g17520	Complementation	ACATGCATGCCGGTTGCAGAATACGTCACT
D570NK572N-F	At2g17520	Mutagenesis	TTCATCGGaACTTGAACCCACAAAA
D570NK572N-R	At2g17520	Mutagenesis	TTTTGTGGGTTCAAGTTCCGATGAA
D590A-F	At2g17520	Mutagenesis	AACTGTCCGCTATGGGCATT
D590A-R	At2g17520	Mutagenesis	AATGCCCATAGCGGACAGTT
N780A-F	At2g17520	Mutagenesis	TCATCAGGGCCAAACTGAAT
N780A-R	At2g17520	Mutagenesis	ATTCAGTTTGGCCCTGATGA

Name	Locus	Purpose	Primer sequences(5'-3')
IRE1A-Ff	At2g17520	Protein expression	GAGGATCCAAAAAGTTTTCGTCGAGGGGCAG
IRE1A-Rf	At2g17520	Protein expression	GACTCGAGTTAGATGATGTCGCATTTGAAGT
pro-BLI-F	At3g23980	Promoter cloning	AGTGGGCCCGAACTGGCAATTCAGAATCGGGAT
pro-BLI-R	At3g23980	Promoter cloning	AGTGGGCCCTGAAAAATACTCGAAATCTCGCAG
scrBLI-F2	At3g23980	Genotyping	AATTGCATGCCATGTTCTTTC
scrBLI-R	At3g23980	Genotyping	ATCTGTTTCCTGGTTGGAGC
scrBLI-F	At3g23980	Genotyping	CCTGCTGCCAATGGAGTAAT
scrBLI-R2	At3g23980	Genotyping	TTTGGGCTTGGATCTGTTGA
scrIRE1AF	At2g17520	Genotyping	CCTTGTTCGATCTCATCATGAGGTT
scrIRE1AR	At2g17520	Genotyping	ACTCGTAACAAGTCACGGATGCTAT
scrIRE1BF	At5g24360	Genotyping	CCTCTCGAACCCTTCAGGTAC
scrIRE1BR	At5g24360	Genotyping	GAAGGAAAACGGACATCCTTC
scrbZIP28F	At3g10800	Genotyping	GTTATAAACCGTGTGGAACCATCGA
scrbZIP28R	At3g10800	Genotyping	CAACTATCTCTCTCACCATGACTAA
scrbZIP60F	At1g42990	Genotyping	CACAGCATCATCGTCTCCTTC
scrbZIP60R	At1g42990	Genotyping	TGGTTCACGTAGTGGGCCATCG
