# Supplementary material for: BLISTER-regulated vegetative growth is dependent on the protein kinase domain of ER stress modulator IRE1A in Arabidopsis thaliana
Source: PLoS Genet. 2019 Dec 23;15(12):e1008563. doi: 10.1371/journal.pgen.1008563 (PMC6946172; doi:10.1371/journal.pgen.1008563)
Supplement: S2 Table — (PDF) [file pgen.1008563.s009.pdf]

Table S2. Parameters for sequencing quality control of each sample.

Sample	Sequences	Clean reads (bp)	Error%	Q20%	Q30%	GC%
WT_1	43139960	6329079936	0.0104	98.3	95.12	46.74
WT_2	65806580	9669480906	0.0101	98.36	95.47	46.66
WT_3	73273118	10766453191	0.0101	98.36	95.47	46.77
*bli*_1	69170248	10167441174	0.0101	98.39	95.55	46.53
*bli*_2	64745858	9506727548	0.0102	98.34	95.42	46.46
*bli*_3	37913350	5559350712	0.0104	98.3	95.13	46.63
*bli ire1a*_1	74192434	10898133626	0.0102	98.35	95.42	46.66
*bli ire1a*_2	48598050	7126936509	0.0104	98.29	95.2	46.82
*bli ire1a*_3	46089842	6762184778	0.0102	98.35	95.33	46.56
*bli ire1b*_1	43831996	6431207944	0.0102	98.34	95.31	46.48
*bli ire1b*_2	48728270	7154566235	0.0102	98.38	95.39	46.62
bli ire1b_3	45463184	6658458885	0.0104	98.27	95.14	46.62
